# Thiazole substitution of a labile amide bond—a new option toward antiplasmodial pantothenamide-mimics

**DOI:** 10.1128/aac.00331-25

**Published:** 2025-08-13

**Authors:** Xiangning Liu, Annica Chu, Mina Nekouei, Chunling Blue Lan, Alexandre Pierret, Karine Auclair, Kevin J. Saliba

**Affiliations:** 1 Research School of Biology, Australian National University 98471, Canberra, Australian Capital Territory, Australia; 2 Department of Chemistry, McGill University 242124, Montreal, Québec, Canada; The Children's Hospital of Philadelphia, Philadelphia, Pennsylvania, USA

**Keywords:** malaria, antimalarial agents, *Plasmodium falciparum*, drug synthesis, drug targets, coenzyme A

## Abstract

The emergence and spread of artemisinin-partial resistant, malaria-causing *Plasmodium falciparum* provide the impetus for developing novel antimalarials. Pantothenamides are potent inhibitors of malaria parasite proliferation; however, their clinical use is hindered by pantetheinase-mediated degradation in human serum. Here, we report the synthesis and biological activity of a series of pantothenamide-mimics in which the thiazole ring replaces the labile amide bond with various orientations. Out of 23 novel compounds generated and tested in the presence of pantetheinase, several display sub-micromolar antiplasmodial activity *in vitro*. A selection of compounds was studied in more detail, and CoA biosynthesis and/or utilization pathways were confirmed to be the target. Toxicity to human cells was not observed. Kinetic studies identified the selected compounds as substrates of the *Hs*PanK3 enzyme, but with much lower affinity compared to that of the natural substrate pantothenate. The most potent thiazole-bearing antiplasmodial compound was found to bind to *Pf*PanK with a 120-fold higher affinity compared to *Hs*PanK, highlighting excellent selectivity, not only against the key first enzyme in the CoA biosynthesis pathway but also at the whole-cell level. In conclusion, thiazole substitution of the labile amide bond represents a promising avenue for developing antimalarial pantothenamide-mimics.

## INTRODUCTION

Malaria is a lethal infectious disease caused by unicellular protozoan parasites of the genus *Plasmodium*. Despite remarkable progress toward malaria elimination, the 2024 World Malaria Report estimated 263 million new cases and 597,000 deaths worldwide attributed to malaria in 2023 (1). Of the six *Plasmodium* species that infect humans (2, 3), *Plasmodium falciparum* is responsible for most of the infections and deaths in the sub-Saharan African region (2, 4). Currently, artemisinin-based combination therapies (ACT) are the first-line treatment for uncomplicated *P. falciparum* malaria in all areas. However, the emergence of ACT-partial resistant parasites and their rapid spread across the globe (1, 5, 6), including in recent cases of severe malaria in Africa (7), threatens the efficacy of ACT, thus hampering the treatment of the disease. To increase the repertoire of available therapeutics, the discovery of new antimalarials with unique mechanisms of action and no cross-resistance with existing drugs is essential (8, 9).

**Fig 1:**
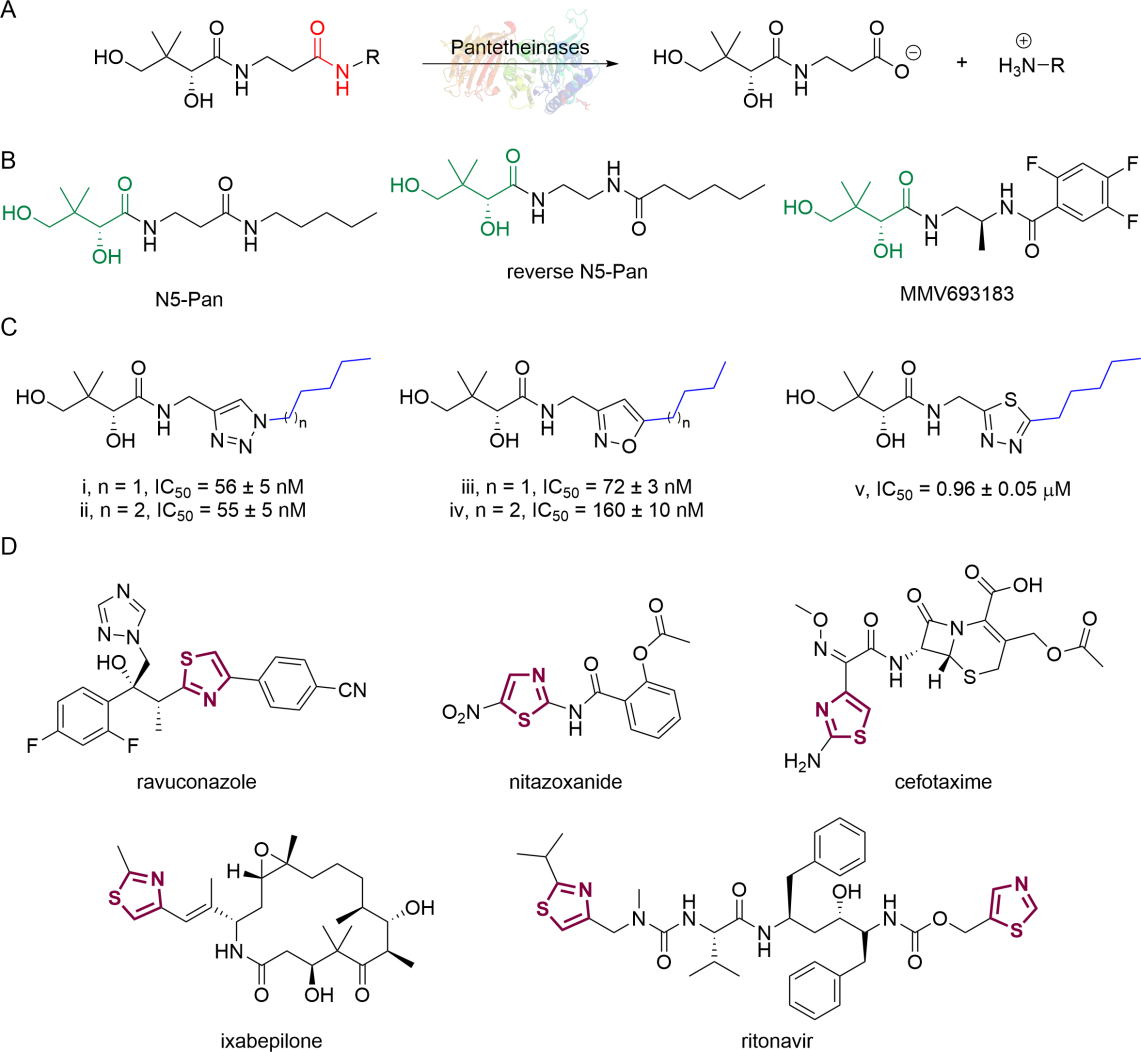
(**A**) Pantothenamides are readily hydrolyzed by serum pantetheinases to yield pantothenate and an amine. The pantetheinase-sensitive amide bond is shown in red. R is commonly an aliphatic or aromatic moiety. (**B**) Structures of N5-Pan, reverse N5-Pan and the preclinical pantothenamide-mimic MMV693183 with the labile amide bond reversed. The pantoyl moiety is drawn in green. (**C**) Structures of compounds **i** to **v** with their respective 50% inhibitory concentrations (IC_50_ values) measured against intraerythrocytic *P. falciparum* in the presence of pantetheinases. Side chains are shown in blue. (**D**) Examples of thiazole-containing pharmaceuticals. Thiazoles are highlighted in maroon.

Water-soluble vitamin B_5_ (pantothenate) is an essential substance required by the intraerythrocytic stage parasites. Pantothenate is the precursor to coenzyme A (CoA), a cofactor involved in many metabolic processes (10, 11). Pantothenate utilization and CoA biosynthesis in *P. falciparum* have been extensively studied (12–15) and have been identified as viable drug targets in the parasite (16, 17). First synthesized by Clifton et al. in 1970 (18), pantothenamides were initially reported to exhibit antibacterial activity (17, 19), with their antiplasmodial activity demonstrated by Spry et al. in 2013 (20). In *P. falciparum*, pantothenamides are first phosphorylated by pantothenate kinase (PanK) and further metabolized by phosphopantetheine adenylyltransferase (PPAT) and dephospho-CoA kinase (DPCK) to generate the corresponding antiplasmodial CoA antimetabolite (21, 22). Despite their high potency and low cytotoxicity *in vitro*, the clinical use of pantothenamides, such as the benchmark compound N5-Pan, is hindered due to their instability *in vivo* (Fig. 1A and B) (20, 23). The pantetheinases in human serum readily hydrolyze the amide depicted in red in Fig. 1A, resulting in the rapid breakdown of pantothenamides. To overcome this issue, our research group and others have employed various structural modifications, including alterations at the *gem*-dimethyl group (24–26), the pantoyl primary alcohol (27), β-alanine (27–30), and the labile amide (27, 29, 31–36) moieties of pantothenamides. In particular, inversion of the labile amide bond has led to very promising compounds, including the discovery of a preclinical pantothenamide-mimic MMV693183 (Fig. 1B) (36). These findings motivated our search for new pantetheinase-resistant pantothenamides.

We have recently focused on replacing the labile amide with heterocycles (32, 33, 35), which are privileged scaffolds in medicinal chemistry. More than 80% of marketed drugs contain at least one heterocyclic fragment in their structure (37, 38). They can participate in diverse secondary interactions due to the presence of the heteroatoms and nuanced aromaticity and can significantly affect the physicochemical properties of the molecules (37, 39). Previously, we reported a series of analogs in which the labile amide group is replaced with diverse heterocycles, resulting in pantothenamide analogs that exhibited pantetheinase resistance and enhanced potency (32, 33, 35). In particular, the 1,4-substituted 1,2,3-triazole with a pentyl or hexyl side chain (compounds **i** and **ii**, Fig. 1C), the 3,5-substituted 1,2-isoxazole with a butyl or pentyl chain (compounds **iii** and **iv**), as well as the 2,5-substituted 1,3,4-thiadiazole (compound **v**), were particularly potent.

Sulfur is a ubiquitous heteroatom in medicinal chemistry (40), and a variety of sulfur-containing scaffolds are employed in the pharmaceutical industry (41). Sulfur is known to serve as an excellent σ-hole to host electrons from adjacent donors, which has a profound impact on conformational stabilization. This can greatly reduce entropic penalty during target engagement (39, 42), thus enhancing potency. One of the most prevalent scaffolds in this category is the thiazole (43). Examples of thiazole-containing pharmaceuticals (Fig. 1D) include ravuconazole (antifungal) (44), nitazoxanide (antiparasitic) (45), cefotaxime (antibacterial) (46), ixabepilone (anticancer) (47), as well as ritonavir (antiviral) (48). Despite the high prevalence of sulfur in medicines, sulfur-containing pantothenamides are currently underdeveloped. To date, several such compounds have been reported (27, 30, 31, 34, 35), but few have had a sulfur-containing heterocycle (35).

Herein, we describe the synthesis, antiplasmodial activity, target determination, and toxicity profile of novel pantothenamide-mimics containing thiazoles. In addition, we report the kinetic analysis of a selection of these compounds using recombinantly expressed and purified human PanK3 (*Hs*PanK3) as well as the *P. falciparum* PanK (*Pf*PanK) in parasite lysates.

## RESULTS

### Synthesis

**Fig 2:**
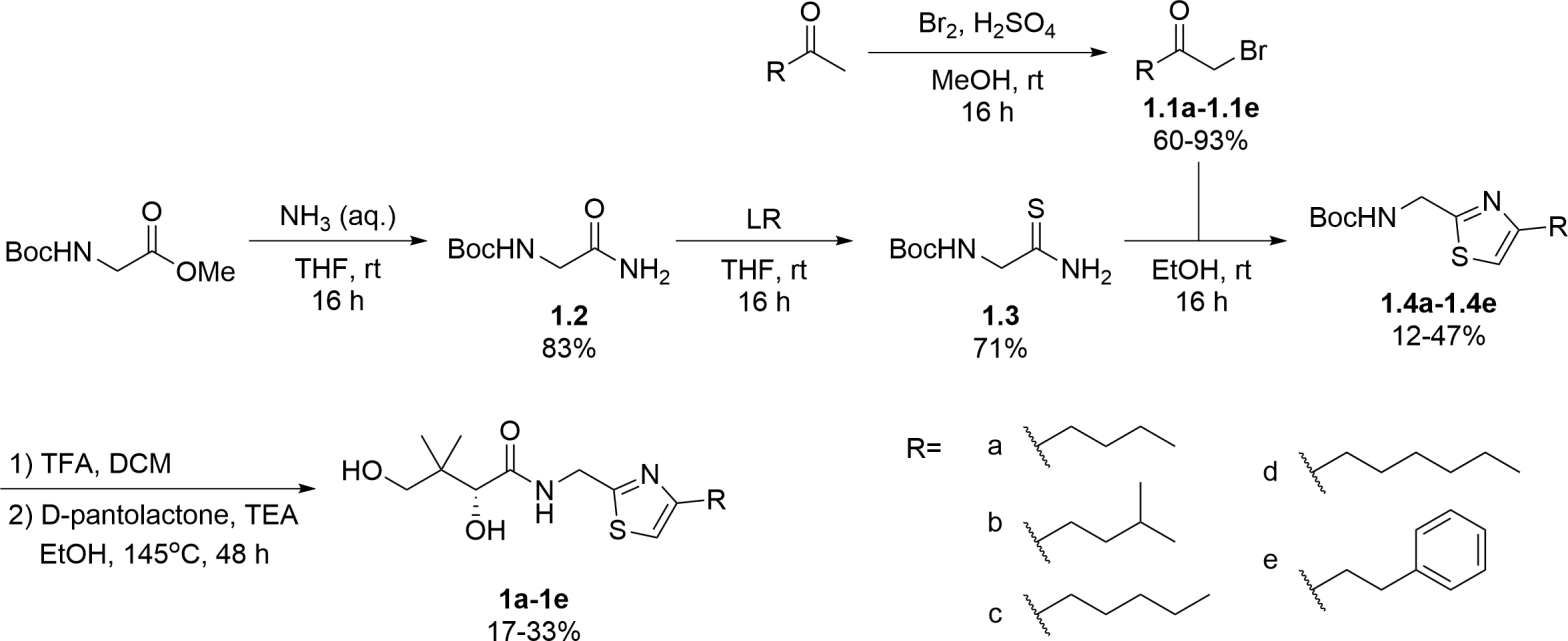
Synthetic route for compounds **1a**–**1e**. Boc: *tert*-butyloxycarbonyl; DCM: dichloromethane; LR, Lawesson’s reagent; rt: room temperature; TEA: triethylamine; TFA: trifluoroacetic acid; THF: tetrahydrofuran.

Considering that a one-carbon linker was reported to be preferred between the pantoyl (in green in Fig. 1B) and the heterocyclic moieties in pantothenamide-mimics containing a triazole or isoxazole in place of the labile amide bond (32, 33, 35), we elected to synthesize thiazole analogs that harbor a one-carbon linker. Figure 2 illustrates the synthetic route for compounds **1a**–**1e** containing 2-aminomethyl-4-substituted thiazoles. Acid-catalyzed monobromination of the methyl ketones was first achieved to access the bromoketone fragments **1.1a**–**1.1e** for Hantzsch thiazole synthesis. The thioamide counterpart was synthesized through an amidation-thiation sequence from Boc-glycine methyl ester. Traditional Hantzsch thiazole synthesis between **1.3** and **1.1a**–**1.1e** generated the thiazole rings. Boc deprotection, followed by aminolysis of D-pantolactone, yielded the target compounds **1a**–**1e**. Notably, the aminolysis conditions under microwave irradiation reported before (31) were not successful here, as decomposition was observed during the reaction. Instead, strictly thermal conditions were employed to avoid decomposition of the starting materials.

**Fig 3:**
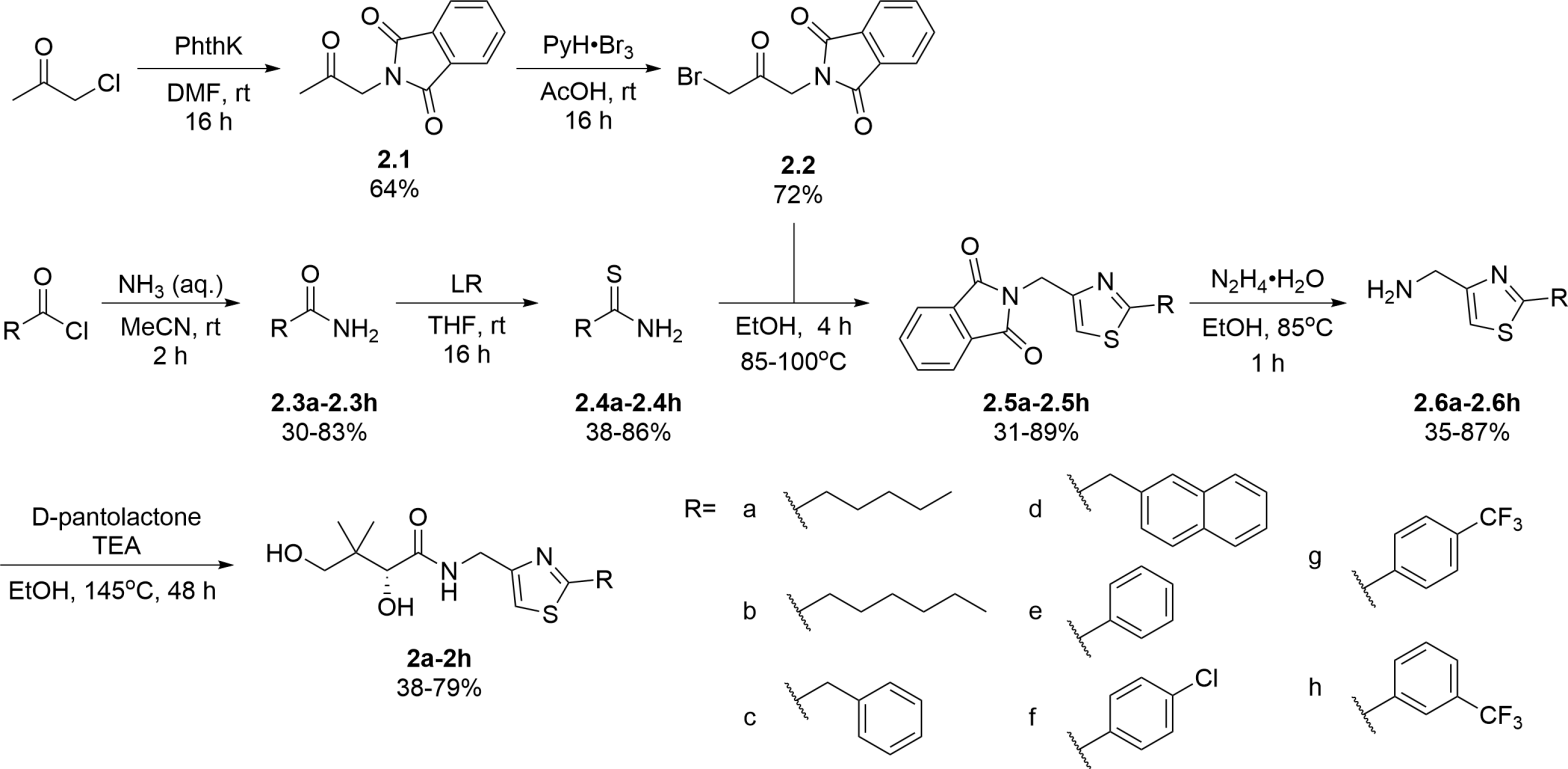
Synthetic route for compounds **2a**–**2h**. DMF: *N, N*-dimethylformamide; LR: Lawesson’s reagent; PhthK: potassium phthalimide; PyH: pyridine; rt: room temperature; TEA: triethylamine; THF: tetrahydrofuran.

A similar approach was adopted for the synthesis of thiazole derivatives with the reverse ring orientation, that is, 4-aminomethyl-2-substituted thiazoles **2a**–**2h** (Fig. 3). Substitution followed by monobromination afforded the bromoketones with a phthalimide-protected amino handle (**2.2**). The same amidation-thiation process as above was used to access the thioamides **2.4a**–**2.4h**, starting from various acyl chlorides. Likewise, Hantzsch thiazole synthesis led to the thiazole core. Finally, removal of the phthalimide protecting group, followed by aminolysis of D-pantolactone, afforded the target compounds.

**Fig 4:**
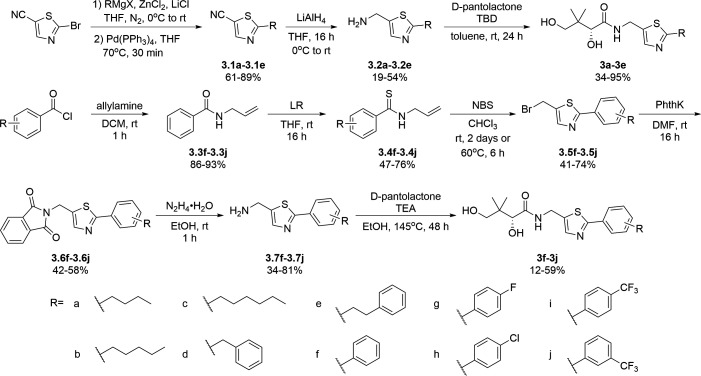
Synthetic route to compounds **3a**–**3j**. DCM: dichloromethane; DMF: *N, N*-dimethylformamide; LR: Lawesson’s reagent; NBS: *N*-bromosuccinimide; PhthK: potassium phthalimide; rt: room temperature; TBD: 1,5,7-triazabicyclo[4.4.0]dec-5-ene; TEA: triethylamine; THF: tetrahydrofuran.

For the synthesis of pantothenamide-mimics with 5-aminomethyl-2-substituted thiazoles (Fig. 4), two different routes were utilized. For synthetic targets harboring a thiazole with alkyl substituents (**3a**–**3e**), decoration of the thiazole was adopted. Thus, Negishi coupling employing 2-bromo-5-cyanothiazole successfully furnished the alkyl-substituted thiazole. Reduction of the nitrile afforded amines **3.2a**–**3.2e**, which were subjected to a recently published method (49) for catalytic D-pantolactone aminolysis to generate the corresponding products. To prepare compounds with an aryl-substituted thiazole (**3f**–**3j**), a reported methodology was followed to access the ring (50). First, the amidation-thiation approach was again utilized to assemble the thioamides **3.4f**–**3.4j**, which underwent *N*-bromosuccinimide-promoted cyclization-oxidation to afford the thiazole core with a bromide handle. Gabriel synthesis converted the bromide handles to amines **3.7f**–**3.7j**, which were subjected to the same thermal aminolysis conditions as above to yield compound **3f**–**3j**.

### *In vitro* antiplasmodial activity

The antiplasmodial activity of all synthetic compounds was evaluated against *P. falciparum*. Thiazoles **1a**, **1e**, and **2e** were found to inhibit intraerythrocytic proliferation of *P. falciparum* with IC_50_ values in the sub-micromolar range (Table 1 and Fig. S1). As evident from the overall higher activity of compound series **1** and series **2** relative to series **3**, it can be concluded that a nitrogen atom is preferred between the substituents of the ring, whereas a sulfur atom is beneficial at the other two ring positions. In series **1**, unlike what was previously reported by Howieson et al. (32) and Guan et al. (33, 35) for triazole- and isoxazole-containing analogs, phenethyl and butyl side chains are equally potent (see **1a** and **1e**), consistent with the length of a phenyl ring (2.8 Å) (51) roughly equaling that of a 3-carbon chain (2.5–2.6 Å) (52). The structure-activity relationships (SARs) reveal that a butyl chain is preferred over a pentyl or a hexyl group, and branching is detrimental. Interestingly, for the 4-aminomethyl-2-substituted thiazole series (**2a**–**2h**), alkyl chains were no longer preferred. Instead, the phenyl group may be favored (as in **2e**) with a slightly lower IC_50_ (0.51 µM) compared to those of **1a** (0.77 µM) and **1e** (0.77 µM), although these did not reach statistical significance at the *P* ≤ 0.05 level (*P* = 0.059 and 0.052, respectively). Moreover, the addition of substituents to the phenyl ring was disadvantageous irrespective of the position, with larger substituents showing a stronger effect, as observed for *para*-chlorine (3.1 µM for **2f**, *P* = 0.0006), *para*-CF_3_ (57 µM for **2g**, *P* < 0.0001), and *meta*-trifluoromethyl (21 µM for **2h**, *P* < 0.0001). A similar trend was observed for the 5-aminomethyl-2-substituted thiazole series (**3a**–**3j**), with the preference for an unsubstituted phenyl group (**3f**) over alkyl groups, despite the overall lower potency of this series. In contrast to what was observed for **2f**–**2h**, **3j** is more potent than **3h**–**3i**, implying that a *meta*-trifluoromethyl group (11 µM for **3j**) is favored over most *para*-substituent group (14–46 µM for **3h**–**3i**, *P* ≤ 0.0038) on the phenyl ring in this series.

**TABLE 1:** Effect of the compounds on the proliferation of intraerythrocytic *P. falciparum*, human foreskin fibroblasts (HFF), and human hepatoblastoma cells (HepG2) at different pantothenate concentrations

Compound		IC_50_ (µM) against *Pf* at 1 µM Pan^*a*^	IC_50_ (µM) against *Pf* at 100 µM Pan^*b*^	Proliferation (%) of HFF with 200 µM Cpd and 16.8 µM Pan^*c*^	Proliferation (%) of HepG2 with 200 µM Cpd and 1 µM Pan^*d*^
1a	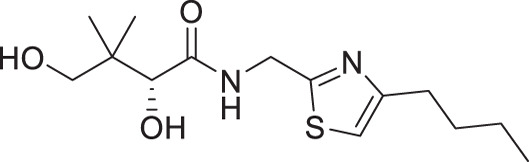	**0.77 ± 0.10**	> 6.25	100 ± 1	71 ± 11
1b	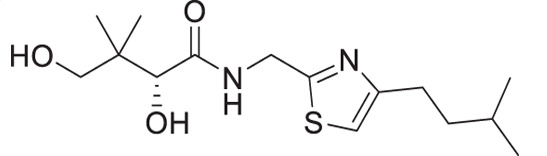	2.9 ± 0.2	-^*e*^	-	-
1c	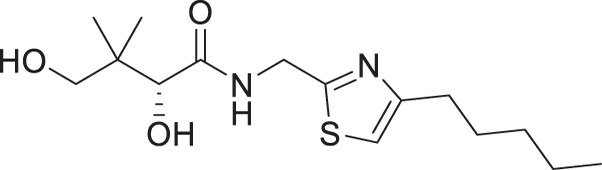	2.3 ± 0.2	> 25	97 ± 4	62 ± 7
1d	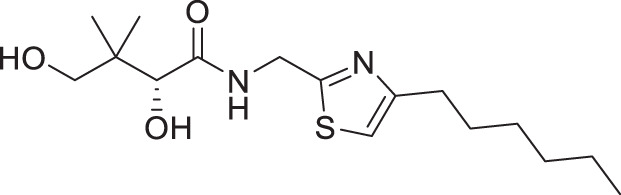	4.3 ± 0.4	-	-	-
1e	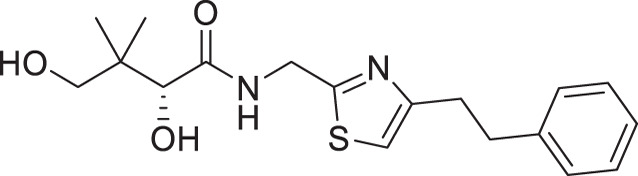	**0.77 ± 0.09**	> 12.5	101 ± 2	55 ± 1
2a	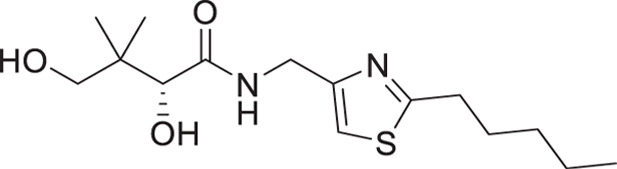	5.6 ± 0.2	-	-	-
2b	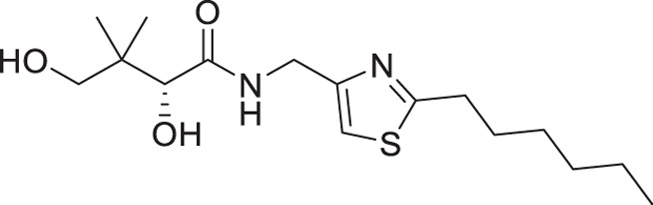	5.2 ± 0.6	-	-	-
2c	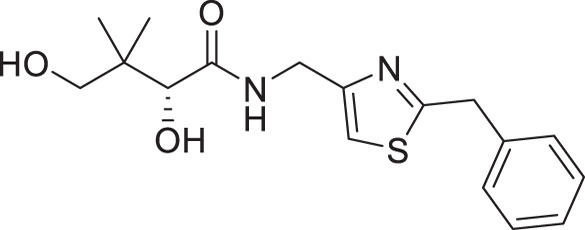	2.4 ± 0.2	> 25	101 ± 2	93 ± 2
2d	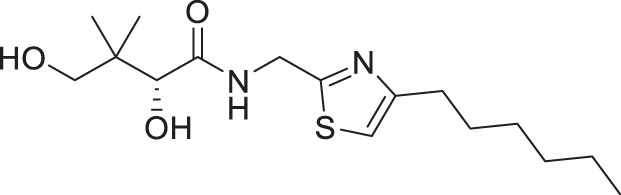	6.5 ± 0.1	-	-	-
2e	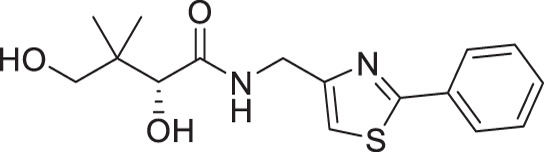	**0.51 ± 0.02**	> 6.25	100 ± 1	91 ± 4
2f	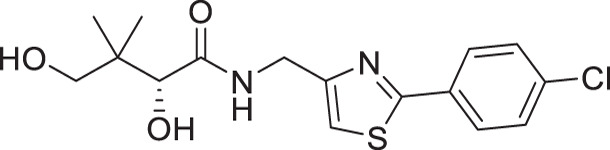	3.1 ± 0.3	-	-	-
2g	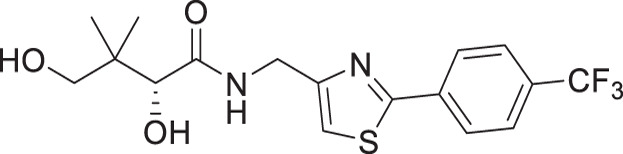	57 ± 2	-	-	-
2h	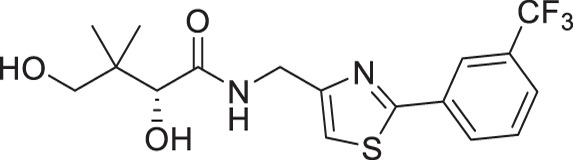	21 ± 1	-	-	-
3a	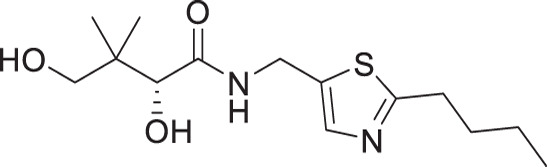	11 ± 1	-	-	-
3b	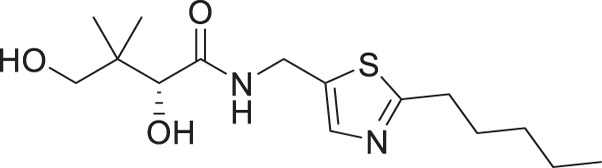	12 ± 1	-	-	-
3c	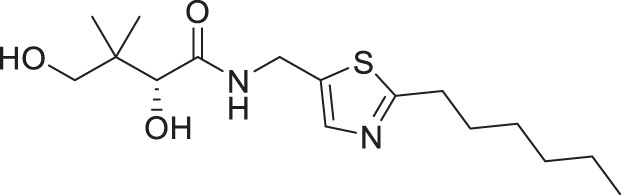	35 ± 2	-	-	-
3d	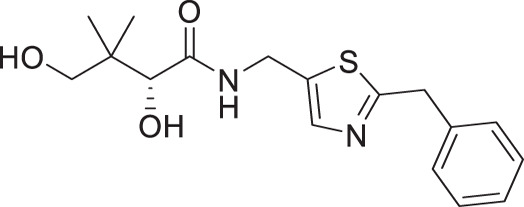	16 ± 1	-	-	-
3e	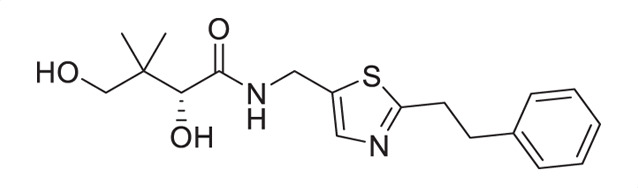	44 ± 1	-	-	-
3f	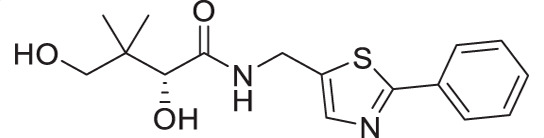	8.0 ± 0.7	> 50	100 ± 1	89 ± 2
3g	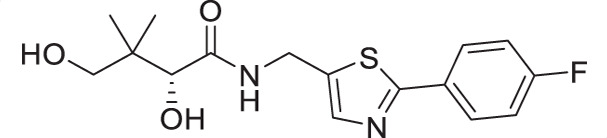	14 ± 2	-	-	-
3h	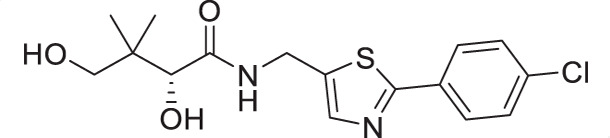	44 ± 4	-	-	-
3i	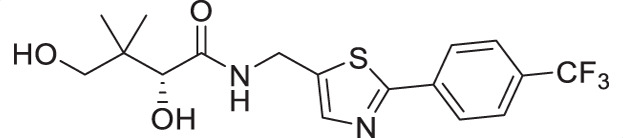	46 ± 6	-	-	-
3j	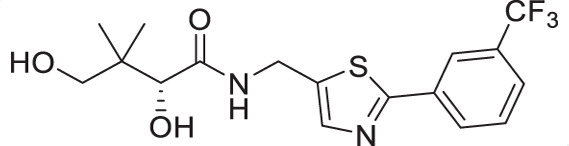	11 ± 1	-	-	-

^*a*^ Antiplasmodial IC_50_ values (µM) were determined against the intraerythrocytic stage of *P. falciparum* (*Pf*) in RPMI-1640 medium containing 1 µM (the normal concentration present in RPMI-1640). IC_50_ values that are sub-micromolar are highlighted in bold.

^*b*^ Antiplasmodial IC_50_ values (µM) were determined against the intraerythrocytic stage of *P. falciparum* (*Pf*) in RPMI-1640 medium containing 100 µM pantothenate.

^*c*^ Human foreskin fibroblast (HFF) proliferation (%) in the presence of 200 µM test compound (Cpd) was determined in DMEM medium containing 16.8 µM pantothenate (the normal concentration present in DMEM).

^*d*^ Human hepatoblastoma cell (HepG2) proliferation (%) in the presence of 200 µM test compound (Cpd) was determined in RPMI-1640 medium containing 1 µM pantothenate. Antiplasmodial data are averaged from three independent experiments, each performed in triplicate, and errors represent SEM. HFF and HepG2 proliferation data are averaged from two independent experiments, each performed in triplicate, and errors represent range/2.

^*e*^ - indicates not determined.

Previously, we have shown that the mechanism of action of heterocyclic pantothenamide-mimics against *P. falciparum* involves bioactivation by PanK, PPAT, and DPCK, resulting in the formation of the corresponding CoA antimetabolites that are hypothesized to inhibit CoA-utilizing enzymes (21, 35), as have been previously observed for reverse pantothenamide-mimics (29, 36). To investigate whether the newly synthesized thiazole derivatives exert their antiplasmodial activity *via* a mechanism that is competitive with pantothenate, the substrate of PanK, we evaluated the antiplasmodial activity of a selection of compounds (**1a**, **1c**, **1e**, **2c**, **2e**, and **3f**) against *P. falciparum* in the presence of a higher concentration of extracellular pantothenate (100 µM, instead of the normal 1 µM that is present in RPMI-1640). Upon adding excess pantothenate, an increase (>6-fold) in IC_50_ values was observed for all tested compounds (Table 1 and Fig. S1), similar to what was previously reported for triazole- and isoxazole-containing pantothenamide analogs (35), indicating that the thiazole derivatives are also competing with pantothenate, most likely as substrates of *Pf*PanK.

Subsequently, the effect of those compounds on the proliferation of human foreskin fibroblast (HFF) cells and human hepatoblastoma cells (HepG2) was assessed using the Incucyte Live-Cell Analysis System, allowing automatic capture of cellular changes and assessment of cell proliferation in the presence of each compound. Cycloheximide (10 µM), a potent protein synthesis inhibitor (53, 54), was used as a positive control, which completely inhibited the proliferation of HFF cells as expected (Fig. S2). By contrast, none of the compounds inhibited the proliferation of the cells at 200 µM, the highest concentration tested, consistent with the compounds being nontoxic to HFF cells (Table 1 and Fig. S2). For HepG2 cells, proliferation was completely inhibited in the presence of 10 µM of puromycin as expected (Fig. S3) (55). By contrast, three (**2c**, **2e,** and **3f**) of the six compounds tested were also completely inactive against HepG2 at 200 µM (i.e*.,* as they were against HFF cells) (Table 1 and Fig. S3). The other three compounds (**1a**, **1c**, and **1e**) showed some low-level toxicity, with only one compound (**1e**) approaching 50% inhibition of proliferation of HepG2 cells at 200 µM (Table 1 and Fig. S3). It should be noted that the HepG2 toxicity screen was carried out in RPMI-1640 (to more closely match the conditions used in the *P. falciparum* assays), and the small degree of toxicity observed for some of the compounds could be due to the lower concentration (1 µM) of pantothenate in the culture medium compared to that present in DMEM (16.8 µM) used for the HFF experiment, rather than a difference in sensitivity by these different cell lines to the compounds.

### Compound interaction with *Hs*PanK3 and *Pf*PanK

**Fig 5:**
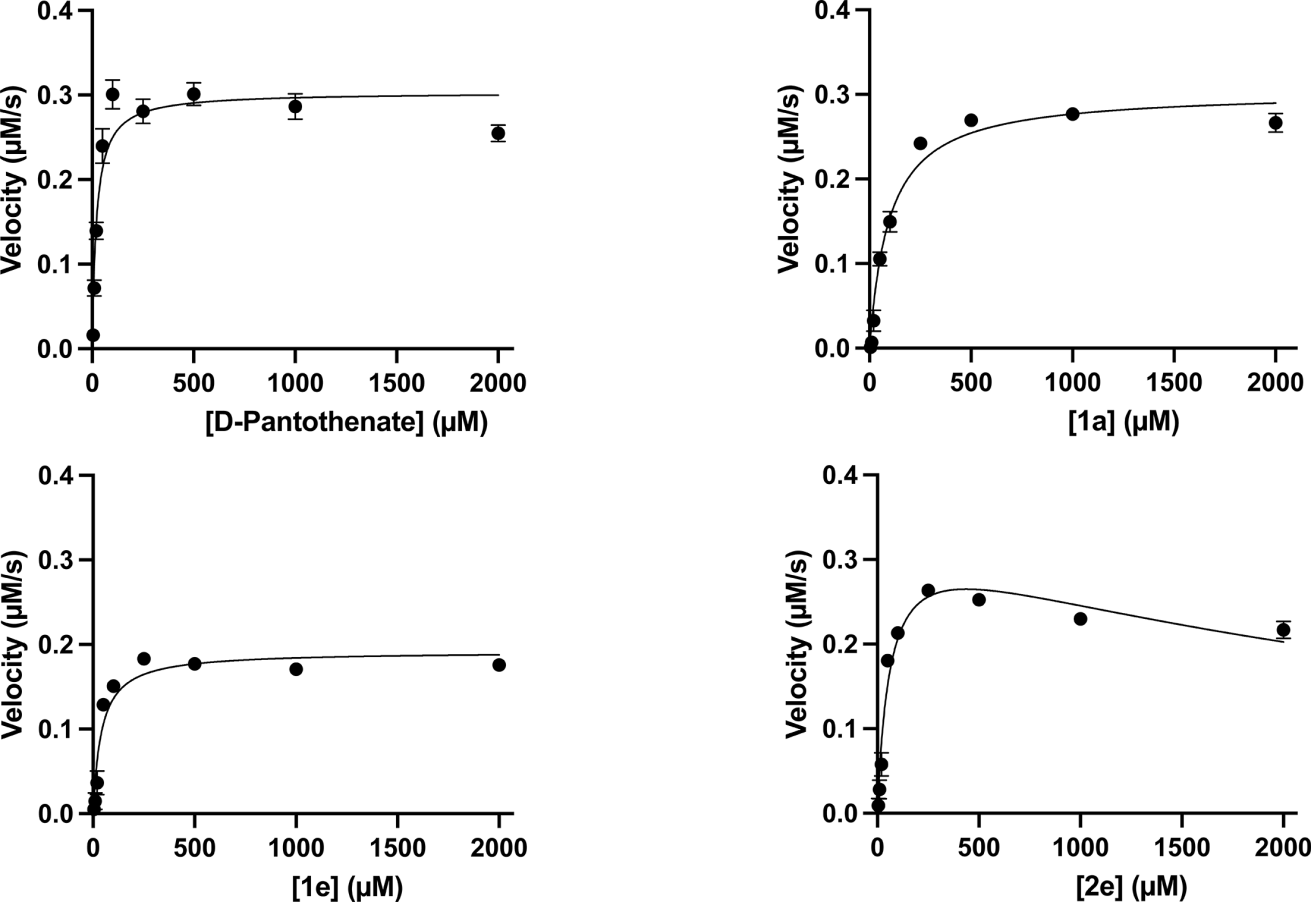
Examples of kinetics plots for *Hs*PanK3 in the presence of representative compounds. Velocities were determined as a function of pantothenate or compound concentration. The data points were fit to the Michaelis–Menten nonlinear regression equation to determine kinetic parameters. For **2e**, Equation 1, which accounts for substrate inhibition, was used to fit the data. Data are averaged from nine experiments, carried out on three different days. Error bars represent SEM and are not visible if smaller than the symbols. Kinetics of other compounds are shown in Fig. S5.

Considering the initial involvement of phosphorylation by *Pf*PanK in the mode of action of the novel thiazole derivatives, kinetic analyses were carried out for selected compounds to compare their transformation by *Pf*PanK and *Hs*PanK3. To date, three phylogenetically distinct types of PanK (type I, type II, and type III) have been characterized, distinguished by their differences in structures, catalytic properties, and inhibition profiles (11, 56). All PanKs characterized to date have been shown to exist as homodimers (57–64), with the exception of those from *P. falciparum* and *Toxoplasma gondii*, another apicomplexan parasite (65). *P. falciparum* expresses two type II PanKs (*Pf*PanK1 and *Pf*PanK2), which have been shown to form a heteromeric complex (65). To explore the difference in activity of the compounds against *P. falciparum* and HFF/HepG2 cells, we set out to perform kinetic analysis of *Hs*PanK3, a well-characterized homodimeric type II PanK expressed in humans, in the presence of selected compounds. *Hs*PanK3 was selected in preference to the other human PanKs on the basis that its cytosolic location (66–69) and feedback inhibition properties (62, 69–71) closely match those of *Pf*PanK (12, 21, 65). *Hs*PanK3 protein was expressed in *E. coli* as a His-tagged protein and purified by affinity chromatography (Fig. S4). The purified *Hs*PanK3 protein was biochemically characterized and had a *K*_m_ for pantothenate of 24 ± 4 µM (*n* = 3; mean ± SEM; Table 2 and Fig. 5), which is consistent with previous reports (17 ± 1 µM) (70, 72). All tested compounds were found to be substrates of *Hs*PanK3. Substrate inhibition, which is a common phenomenon in enzyme kinetics (73–75), was observed for compounds **2c** and **2e** (Fig. 2 and 5). Such inhibition is typically caused by simultaneous binding of two or more substrate molecules to the enzyme, resulting in the formation of an unproductive enzyme–substrate complex (76). This increases with rising substrate concentration. Equation 1 (Y = V_max_*X/(*K*_m_ + X*(1 + X/*K*_i_))), X: substrate concentration, Y: enzyme activity, V_max_: maximum enzyme activity, *K*_m_: Michaelis–Menten constant, and *K*_i_: inhibitor constant), which accounts for substrate inhibition, was used to determine the kinetic parameters of **2c** and **2e**, instead of the standard Michaelis–Menten equation that was used for the other compounds. We found that *Hs*PanK3 exhibited a 1.8-fold to 4.2-fold higher *K*_m_ (i.e.*,* lower affinity) for all selected compounds compared to that of the natural substrate pantothenate (*P* ≤0.003). By contrast, the turnover numbers (*k*_cat_) of *Hs*PanK3 for **3f** were slightly higher than that of pantothenate (i.e.*,* higher efficiency, *P* = 0.0059). The *k*_cat_ for other compounds was the same as (**1a**, **1c**, **2c**, and **2e**) or slightly lower (**1e**, *P* <0.0001) than the *k*_cat_ for pantothenate. The specificity of *Hs*PanK3 for each compound was then evaluated by computing the *k*_cat_/*K*_m_ values. In the presence of the compounds with sub-micromolar antiplasmodial activity (**1a**, **1e,** or **2e**), *Hs*PanK3 exhibited a 2.5-fold to 4.5-fold lower *k*_cat_/*K*_m_ (i.e., lower specificity) compared to the natural substrate pantothenate (*P* < 0.0001; Table 2). Similar results were obtained for **1c**, **2c**, and **3f** (~3-fold lower, *P* < 0.0001), consistent with *Hs*PanK3 having a much higher catalytic efficiency for pantothenate than for the thiazole analogs. Together with the lack of toxicity observed on HFF and HepG2 cells, these data imply that the compounds may not be substantially metabolized ππby the human enzyme.

**TABLE 2:** Kinetic data calculated for *Hs*PanK3 in the presence of various pantothenamide-mimics^*a*^

Compound		*K*_m_ (µM)	*k*_cat_ (s^−1^)	*k*_cat_ / *K*_m_ (mM^−1^ s^−1^)	*K*_i_ (µM)^*c*^
D-Pantothenate	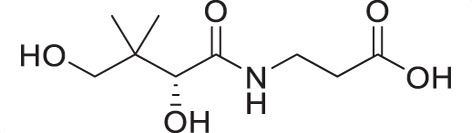	24 ± 4	0.64 ± 0.02	30 ± 3	**-**
1a	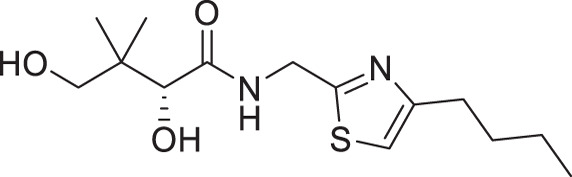	100 ± 7	0.64 ± 0.01	6.6 ± 0.4	**-**
1c	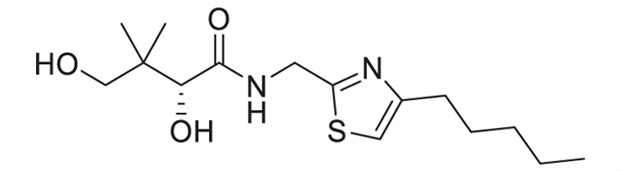	65 ± 7	0.61 ± 0.02	10.2 ± 0.9	**-**
1e	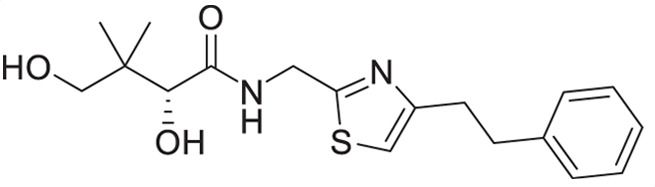	42 ± 4	0.399 ± 0.005	10 ± 1	**-**
2c^*b*^	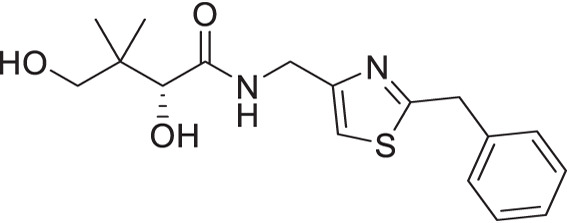	69 ± 6	0.63 ± 0.02	9.3 ± 0.5	2,654 ± 733
2e^*b*^	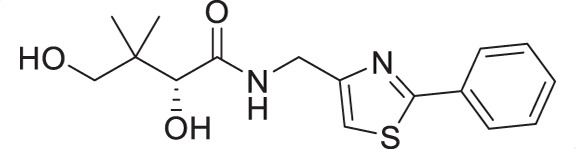	67 ± 10	0.73 ± 0.05	12 ± 1	4,337 ± 961
3f	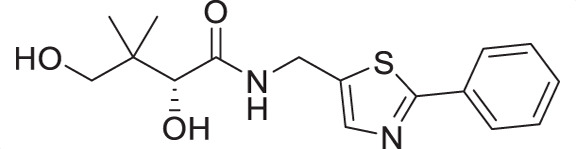	75 ± 11	0.73 ± 0.02	10.7 ± 0.8	-

^*a*^ Unless otherwise stated, kinetic parameters reported in the table were determined for each experiment by fitting the Michaelis–Menten equation to the data.

^*b*^ For these compounds, the parameters were determined by fitting the data to equation 1 that accounts for substrate inhibition.

^*c*^ *K_i_*, the inhibitor constant, was determined for compounds that displayed substrate inhibition, that is, a decrease in *V*_max_ at higher substrate concentrations. Data were collected from three independent experiments, each performed in triplicate (errors represent SEM).

**Fig 6:**
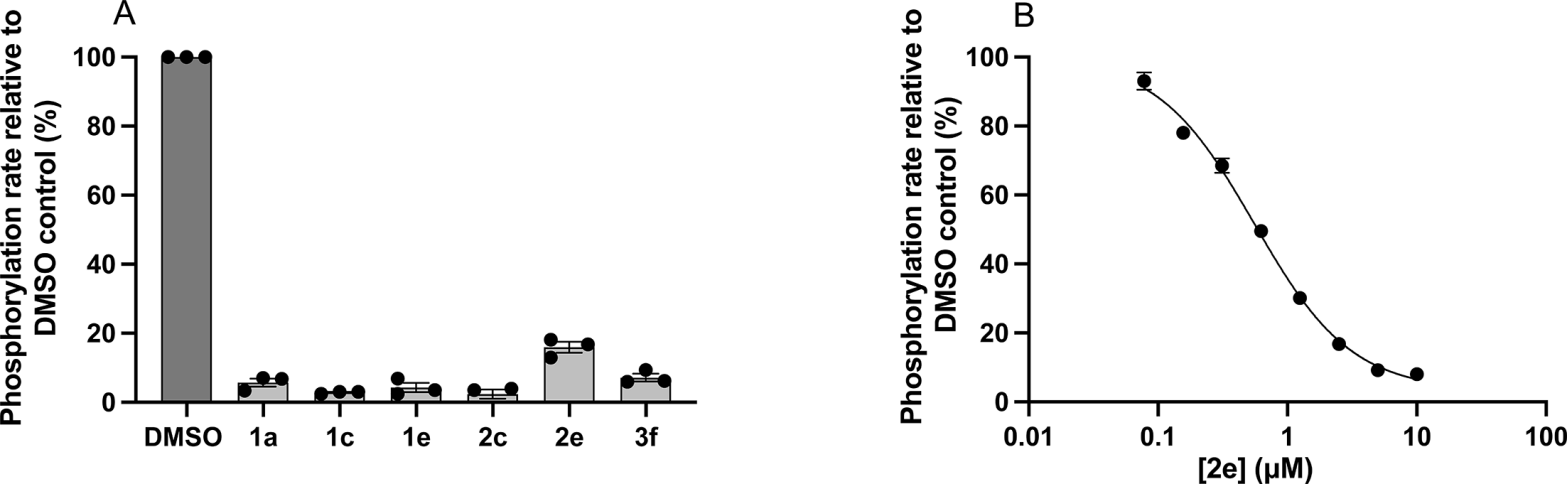
Effect of various compounds on [^14^C]pantothenate phosphorylation by *P. falciparum* lysates. [^14^C]pantothenate and Somogyi reagent were used to detect PanK activity in *P. falciparum* lysates. Compounds were tested at 10× their antiplasmodial IC_50_ values in (**A**). The effect of **2e** on [^14^C]pantothenate phosphorylation by *P. falciparum* lysates was tested at various concentrations in (**B**). Data are expressed as a percentage of the pantothenate phosphorylation rate in the DMSO (vehicle) control and are averaged from three independent experiments, each with a different batch of lysate and carried out in triplicate. Error bars represent SEM and are not visible if smaller than the symbols.

Additional experiments were performed with the same selected compounds to further investigate their antiplasmodial mechanism(s) of action. We are specifically interested in determining whether there is a correlation between the antiplasmodial activity of the compounds and their interaction with *Pf*PanK, as we have shown that the thiazole derivatives compete with pantothenate (Table 1, Fig. S1). Although we would like to directly interrogate the kinetic parameters of *Pf*PanK in the presence of each compound, the low yields achievable when purifying the enzyme from *P. falciparum* prohibited us from obtaining enough protein for conventional kinetic analysis. As an alternative, we tested the phosphorylation of [^14^C]pantothenate by *Pf*PanK in *P. falciparum* lysates in the presence of each compound at 10× their respective antiplasmodial IC_50_ values (Table 1, Fig. 6A). Almost complete inhibition (>90%) of pantothenate phosphorylation was observed in the presence of 10× antiplasmodial IC_50_ values of **1a**, **1c**, **1e**, **2c**, and **3f**, while approximately 80% inhibition was observed for compound **2e**, which adds another line of evidence that these compounds are competing with pantothenate for direct interaction with *Pf*PanK.

To examine the interaction of compound **2e**, the most potent antiplasmodial in this series, with *Pf*PanK in more detail, we tested the effect of various concentrations of **2e** on [^14^C]pantothenate phosphorylation. As expected, we observed a reduction in the pantothenate phosphorylation rate by *Pf*PanK (in lysates) as the concentration of **2e** was increased (Fig. 6B). We found that **2e** was equally effective (*P* = 0.256) at inhibiting blood stage parasite proliferation (IC_50_ = 0.51 ± 0.02 µM; *n* = 3; mean ± SEM) and pantothenate phosphorylation (IC_50_ = 0.56 ± 0.03 µM; *n* = 3; mean ± SEM). The IC_50_ of **2e** at inhibiting pantothenate phosphorylation is approximately 2.1-fold higher (*P* = 0.046) than the *K*_m_ for pantothenate that has been reported previously for *Pf*PanK in parasite lysates (270 ± 95 nM; mean ± SEM) (21, 77, 78). In comparison, *Hs*PanK3 exhibits a 2.8-fold higher *K*_m_ for **2e** (67 ± 10 µM; *n* = 3; mean ± SEM); thus, the IC_50_ of **2e** at inhibiting *Pf*PanK-mediated pantothenate phosphorylation is 120-fold lower (*P* = 0.0032) than the *K*_m_ observed for *Hs*PanK3 (Table 2), consistent with **2e** interacting with much higher affinity with *Pf*PanK than *Hs*PanK3, and with the absence of toxicity to HFF and HepG2 cells.

### Computational analysis of pantothenamides

The mode of action of pantothenamides may involve three bioactivation steps and several CoA-utilizing enzymes; therefore, small structural differences can affect permeability (across both erythrocyte and parasite membranes) as well as their binding to any of the many enzymes that are involved in bioactivation and/or serve as targets. It is therefore challenging to rationalize, for example, the preference for a phenethyl or phenyl group over the linear aliphatic chains observed for series **2** and **3** compounds. Further experiments specifically looking at cell permeability, bioactivation, and target identification might help gain a better understanding of the SARs. As a complementary approach, ligand-based computational studies were used here to gain more insight into SARs. Conformational samplings and electronic charges were calculated using ORCA version 6.0 (79, 80). The results were visualized in ChimeraX (81–83) with SEQCROW (84, 85). Conformational samplings were obtained from the semi-empirical tight binding (GFN2-xTB) method (86) and the analytical linearized Poisson–Boltzmann (ALPB) model (87) as an implicit solvent model of water. Electronic charge distributions were calculated based on charges from electrostatic potentials using a grid-based method (CHELPG) (88) at the B3LYP/def2-TZVPPD level of theory.

**Fig 7:**
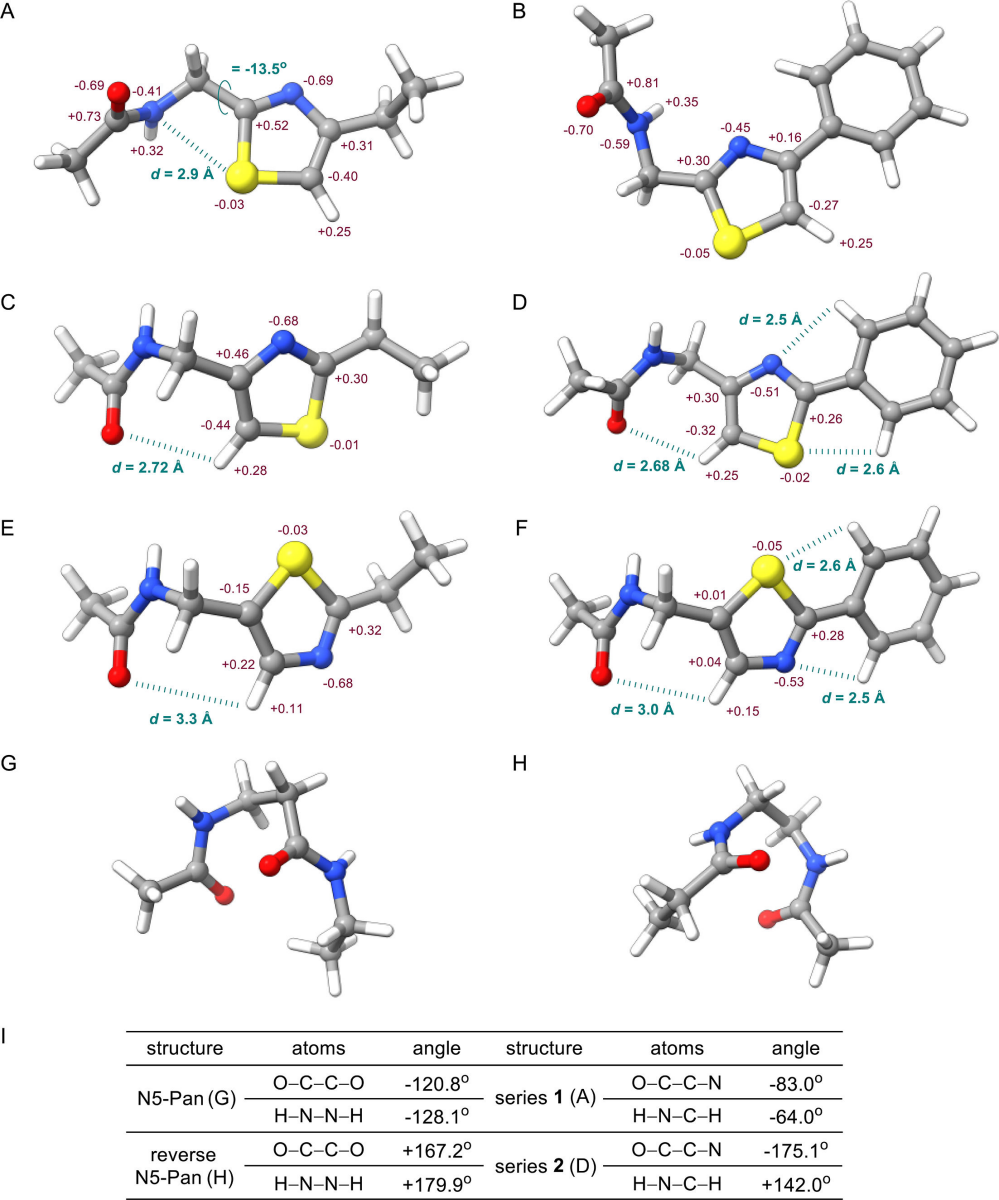
Computational results. Distances and torsion angles are highlighted in teal, and charge distributions are indicated in maroon. The global minimum conformations are shown, except for structure b, which is +0.43 kcal/mol above the global minimum. (**A**) Truncated model structure of series **1** bearing an ethyl substituent. (**B**) Truncated model structure of series **1** bearing a phenyl substituent. (**C**) Truncated model structure of series **2** bearing an ethyl substituent. (**D**) Truncated model structure of series **2** bearing a phenyl substituent. (**E**) Truncated model structure of series **3** bearing an ethyl substituent. (**F**) Truncated model structure of series **3** bearing a phenyl substituent. (**G**) Truncated model structure of N5-Pan bearing an ethyl substituent. (**H**) Truncated model structure of reverse N5-Pan bearing an ethyl substituent. (**I**) Measured torsion angles.

We focused on the different thiazole cores and simplified the shared structures of the compounds. Consequently, the pantoyl moiety was reduced to an acetyl group, and the side chain (defined in Fig. 1) was abbreviated with an ethyl or a phenyl substituent. Overall, both the position of the heteroatoms in the ring and the nature of the side chain were found to have a significant impact on the favored conformations and the electronic charges. For series **1** with an ethyl substituent, conformational sampling reveals that a 1,4 N···S interaction is key to the stabilization of the amide-thiazole backbone (Fig. 7A). Taking a closer look at the global minimum of the structure, the distance between the nitrogen and sulfur atom is 2.9 Å, which is within the sum of the van der Waals radii of 3.35 Å (41). The torsion angle (φSCCN) is −13.5 degrees, which satisfies the requirement for 1,4 N···S interaction (41). Notably, the lesser negative charge on the amide nitrogen in the ethyl model (−0.41) than in the phenyl model (−0.59; Figure 7B) correlates with the proposal that this nitrogen serves as an electron donor in the 1,4 N···S interaction. Charge distribution analyses also show that the ring N−C−C−H moiety in series **1** with an ethyl substituent is polarized, similar to that of an amide O−C−N−H moiety. Conversely, a phenyl substituent (rather than ethyl) is detrimental in this series (Fig. 7B). Despite slightly improving the conformational stability of the ensemble (see section 4 of the Supporting Information), installation of a phenyl group not only disrupts the 1,4 N···S interaction but also makes the N−C−C−H moiety less polar.

As sulfur is positioned away from the amide bond in series **2**, no benefit can be gained from a 1,4 N···S interaction (Fig. 7C D). Nevertheless, the different arrangement of the heteroatoms offers new favorable interactions. Unlike series **1**, in series **2,** the introduction of a phenyl substituent (Fig. 7D) significantly stabilizes the conformational ensemble (see section 4 of Supporting Information), consistent with the higher activity of **2e** over that of **2a**. In particular, the thiazole core and the phenyl ring are essentially coplanar across the whole ensemble, implying that they form an important interaction. Previous studies (89–91) have suggested that this phenomenon originates from unconventional hydrogen bonds between the heteroatoms and the aromatic C-H bond (C−H···N and C−H···S hydrogen bond). Indeed, the distance between N/S and H calculated here is 2.5 Å and 2.6 Å, respectively, therefore within the sum of the van der Waals radii (2.75 Å and 3.0 Å) and confirming this beneficial interaction. A polarized N−C−C−H thiazole backbone is also present in series **2**, indicating its capability of electronically imitating the amide bond of pantothenamides. In addition, the distance between the amide oxygen and thiazole hydrogen is slightly below the sum of the van der Waals radii (2.72 Å), suggesting that extra factors may contribute to conformational stability. This may explain, at least in part, the better activity of series **1** and **2** relative to previously reported linear pantothenamides, such as N5-Pan, for which the IC_50_ values are 124 ± 15 µM and 2.0 ± 0.5 µM in the presence and absence of pantetheinases, respectively (20).

Results with series **3** compounds somewhat resemble those of series **2**, albeit with less beneficial interactions (Fig. 7E and F), thus paralleling their lower biological activity. The 1,4 N···S interaction is absent in series **3**, and the amide oxygen and thiazole hydrogen are too far apart (3.3 Å and 3.0 Å for the ethyl and phenyl derivatives, respectively) to have any meaningful interactions. Remarkably, charge distribution analysis reveals that no polarized thiazole backbone exists in series **3**, making it electronically unfit to mimic an amide bond. These results correlate well with the fact that series **3** is generally inferior to series **1** and **2** for their antiplasmodial activity. They also tentatively offer a plausible explanation for the different substituent preferences among these series of compounds. For example, the phenyl-thiazole interaction may rationalize the better activity of **3f** over that of **3a** in this series too.

Although both series **1** and **2** delivered potent antiplasmodial compounds, they had very distinctive SARs. To further explain the difference between the observed SARs, conformational samplings were also computed for the benchmark compounds N5-Pan and reverse N5-Pan. Similar reduction of molecular complexity was applied to facilitate the calculations. The results demonstrate that both compounds exhibit a U-shaped backbone to maximize amide carbonyl–carbonyl interactions, either through $n\to \pi^{}$^*^ interactions or dipole–dipole interactions (92–94). Analyses of torsion angles were performed to compare amide bonds and the polarized thiazole backbones, where angles of O−C−C−O and H−N−N−H were measured for the two pantothenamides, and angles of O−C−C−N and H−N−C−H were measured in thiazole-containing pantothenamide-mimics. Gratifyingly, the results (Fig. 7) revealed that series **1** bears more resemblance to the normal amide, whereas series **2** with the reverse amide. This correlates well with previous findings that alkyl chains were preferred for compounds with a normal amide, whereas aryl substituents were favored for those with a reverse amide (20, 29, 36, 95).

## DISCUSSION

A goal of this study was to explore the antiplasmodial activity, specificity, and on-target activity of pantothenamide-mimics, which have the labile amide bond replaced with a thiazole to prevent degradation by pantetheinases. We previously had success at replacing this bond with a triazole or an isoxazole (Fig. 1C). Preliminary results (35) suggested that a sulfur-containing heterocycle could be a promising replacement for the labile amide bond; hence, we were encouraged to synthesize novel thiazole analogs. Three of the 23 compounds displayed sub-micromolar antiplasmodial activity against blood stage-*P. falciparum* in the presence of an extracellular pantothenate concentration of 1 µM, a concentration that is similar to the normal human plasma pantothenate concentration (1.6–2.7 µM [96]).

In the pantothenamide-mimics previously reported by Spry et al*.* (30), Schalkwijk et al*.* (29), Guan et al*.* (33, 35), and de Vries et al*.* (36), a two-carbon linker is optimal between the pantoyl and the linear amide mimic, whereas a one-carbon linker was preferred between the pantoyl and the heterocyclic moieties. Therefore, the novel thiazole analogs reported here all harbor a one-carbon linker. The substituent preferences at the ring or amide nitrogen also differ between analogs containing a heterocycle and those with a linear amide. In the case of linear amide mimics, substituents such as phenyl or phenethyl are preferred over linear aliphatic groups (28–30). On the other hand, linear aliphatic chains with a length of C4 to C6 are preferred over other alkyl or aryl groups (e.g.*,* 2-methyl-propyl, *p*-trifluoromethylphenyl, phenethyl groups) for molecules containing a triazole or isoxazole (32, 33, 35). By contrast, we found that phenethyl and butyl side chains give equally potent compounds in the thiazole series **1** (see **1a** and **1e**). Surprisingly, in the thiazole series **2** and **3**, the alkyl chains are no longer preferred, and a phenyl group is favored instead (see **2a** and **2e**; **3a** and **3f**), yet substituents on the phenyl ring are detrimental. Preliminary computational studies were used to rationalize these SARs. A ligand-based approach was used, considering the complex mode of action of these molecules and the several steps needed for their localization into the parasite. The results confirm that both the geometry of the heterocycles and the position of heteroatoms have a considerable impact on the side chain preference and thus on their biological activities. This was consistent with previous findings that isosteric heterocycles showed a wide range of activities. The rationale behind the compounds in this study was tentatively explored by the conformational sampling as well as charge analysis of the molecules. Overall, this explains why the biological activity of pantothenamide mimics is so easily tuned, which is highly advantageous in drug development.

Kinetic analyses with *Hs*PanK3 revealed that the tested thiazole analogs are substrates of the enzyme, yet with lower specificity than pantothenate, as manifested by the higher *K*_m_ and lower *k*_cat_/*K*_m_ values. For comparison, we employed a [^14^C]pantothenate-based phosphorylation assay to investigate the effect of selected compounds on pantothenate phosphorylation in parasite lysates. This radiolabeled assay is unable to distinguish whether a compound is a substrate or an inhibitor, but it can show whether the compound competes with pantothenate for binding to *Pf*PanK. However, based on the *Hs*PanK3 data presented above and a previous report that demonstrated that a similar triazole derivative (compound **i**, Fig. 1C) is a *Pf*PanK substrate (21), we hypothesize that the compounds tested here are *Pf*PanK substrates, rather than inhibitors. We show that the most active compound (**2e**) is equally effective at inhibiting the phosphorylation of pantothenate by *Pf*PanK than at preventing the proliferation of intraerythrocytic *P. falciparum* parasites, yet we are not proposing that *Pf*PanK is the target of **2e**, or more generally, the target of this series of compounds. Instead, it is likely that the target is downstream in the CoA biosynthetic pathway and/or a CoA-utilizing enzyme. This is consistent with previous reports on the triazole and reverse amide pantothenamide-mimics (21, 29, 32, 36). The latter were reported to be metabolized into CoA antimetabolites, which target and inhibit acetyl-CoA synthetase (29, 36). Our data are consistent with the thiazole-containing pantothenamide-mimics sharing a similar mechanism of action as the previously reported ones.

Remarkably, our results with HFF and HepG2 cells suggest that the compounds may not be cytotoxic to mammalian cells. Considering the fact that the compounds are substrates for *Hs*PanK3, one explanation for the lack of toxicity against HFF and HepG2 cells may be that the compounds are not able to permeate the cells, as previously hypothesized by Howieson et al*.* (32) for HFF cells. Alternatively, the compounds may be phosphorylated by human PanK but not metabolized further by downstream enzymes of the CoA biosynthetic pathway in HFF/HepG2 cells (and/or may be rapidly hydrolyzed), as opposed to in *P. falciparum*, where their phosphorylated form subsequently rejoins the pathway as substrates of *Pf*PPAT and *Pf*DPCK, generating antiplasmodial CoA antimetabolites (21, 22). If indeed these compounds exert their activity by inhibiting CoA-utilizing enzymes, they would be ineffective in HFF and HepG2 cells due to the lack of production of the CoA antimetabolites. An alternative explanation is that HFF or HepG2 cells do generate the CoA antimetabolites, but they are ineffective at inhibiting downstream CoA-utilizing enzymes. Additional work is required to differentiate between these possibilities.

We propose that replacing the labile amide group of pantothenamides with a thiazole ring is a promising strategy to identify blood-stable antiplasmodial agents targeting CoA metabolism/utilization. Additional SAR studies, in particular at the side chain of both thiazole series **1** and **2**, and further biological testing are worthy of investigation.

## MATERIALS AND METHODS

### Cell culture

Culturing of intraerythrocytic stage *P. falciparum* parasites (3D7 strain) was carried out using a method adapted from Trager and Jensen (97) and Allen and Kirk (98). Parasites were maintained within human, male or female, erythrocytes (typically Group O+, Rh+, 4% hematocrit) in RPMI-1640 medium (Thermo Fisher Scientific, catalogue number 72400120) supplemented with 25 mM HEPES, 11 mM glucose, 200 µM hypoxanthine, 24 µg/mL gentamicin and 6 g/L Albumax II on a horizontally rotating shaking incubator (New Brunswick Innova 42/42R) at 37°C under an atmosphere of 96% nitrogen, 3% carbon dioxide, and 1% oxygen.

HFF cells were maintained *in vitro* in DMEM supplemented with 10% (vol/vol) newborn calf serum, 50 units/mL penicillin, 50 µg/mL streptomycin, 10 µg/mL gentamicin, 0.2 mM L-glutamine, and 0.25 µg/mL amphotericin B, as previously described (99). HepG2 cells were maintained in RPMI-1640 medium (Thermo Fisher Scientific, catalogue number 72400120) supplemented with 10% (vol/vol) fetal bovine serum, 50 units/mL penicillin, and 50 µg/mL streptomycin.

### *In vitro* parasite proliferation assay

To evaluate the effect of each compound on the *in vitro* proliferation of *P. falciparum* parasites, a SYBR Safe-based fluorescence assay was used as previously described (32). All pantothenamide-mimics tested were initially dissolved to a concentration of 200 mM in DMSO. The final DMSO concentration in the assay never exceeded 0.1% (vol/vol). Assays were performed using the same medium used to culture the parasites. Where specified, 100 µM sodium pantothenate (Yick-Vic Chemicals and Pharmaceuticals, Hong Kong) was added to the medium. Assays were set up with synchronous ring-stage *P. falciparum*-infected erythrocytes in 96-well microtiter plates. Parasites were synchronized using 5% (wt/vol) sorbitol (100). Two-fold serial dilutions of the compound in complete medium were then added to the wells of each plate in triplicate (total volume of 100 µL per well), after which *P. falciparum*-infected erythrocytes were added to a final volume of 200 µL per well (0.5% parasitemia and 1% hematocrit). *P. falciparum*-infected erythrocytes incubated in the presence of 0.5 µM chloroquine were used as a non-proliferation control, and those incubated in the absence of compounds and chloroquine served as a 100% parasite proliferation control. To minimize “edge effects” (101, 102), the outermost rows and columns of the microtiter plates were not used in the proliferation assays and were filled with 200 µL of medium. The plates were then incubated at 37°C for 96 h under a low-oxygen atmosphere. At the end of the incubation, plates were frozen at −80°C to lyse the cells. Following thawing, the content of each well was resuspended in the plates by pipetting, and then 100 µL from each well was transferred to a second 96-well microtiter plate containing 100 µL of lysis buffer (20 mM Tris, pH 7.5; 5 mM EDTA; 0.008% [wt/vol] saponin and 0.08% [vol/vol] Triton X-100) with SYBR Safe DNA gel stain (0.2 µL/ml) per well. The cells were gently mixed with the buffer, and the fluorescence signal in each well was then measured using a FLUOstar OPTIMA microplate reader with excitation and emission wavelengths of 490 and 520 nm, respectively, after setting the gain on a 100% parasite proliferation control well. The fluorescence in the non-proliferation control wells was subtracted from the fluorescence readings in the other wells prior to further analysis. Parasite proliferation, calculated as a percentage of the fluorescence measured in the test wells relative to the fluorescence measured in the 100% parasite proliferation control wells, was then plotted against the logarithm of the compound concentration. Using GraphPad Prism, each data set was then fitted with a sigmoidal curve (Y = Bottom + (Top-Bottom)/(1+(IC_50_/X)^HillSlope), where Y represents percentage parasite proliferation, IC_50_ represents the concentration of the compound resulting in 50% parasite proliferation, and X represents the compound concentration) using a nonlinear least squares regression. No constraints were used when curve fitting for antiplasmodial assays were performed in the presence of 1 µM pantothenate. The bottom of the curve was constrained to 0 when fitting for antiplasmodial assays were performed in the presence of 100 µM pantothenate. IC_50_ values (determined from the sigmoidal curves) were averaged from three independent experiments.

### *In vitro* HFF and HepG2 proliferation assay

*In vitro* HFF proliferation assays were performed as previously described by Howieson et al*.* (32), with modifications. Confluent HFF cells (in a 75 cm^2^ flask) were harvested by treatment with 1 × trypsin–EDTA (0.25% [wt/vol] trypsin +0.2 g/L EDTA) and diluted in 21 mL of DMEM supplemented with 10% [vol/vol] newborn calf serum (32). Assays were set up in 96-well microtiter plates in a similar way to that described for the antiplasmodial activity assay. Cycloheximide (10 µM) was used as a non-proliferation control, and 0.1% (vol/vol) DMSO served as a 100% proliferation control. The plates were incubated at 37°C in a CO_2_ incubator for 96 h, and the Incucyte Live-Cell Analysis System (Essen BioScience) was used to capture cellular changes and assess cell growth in each well over time. Each well was scanned to capture bright field images, and confluency was assessed at the end of the 96 h incubation, which was then used to evaluate the effect of each compound on the proliferation of HFF cells by comparing it to the 100% proliferation control. The bottom of the curve was constrained to 0 when fitting for HFF proliferation assays using GraphPad Prism.

*In vitro* HepG2 proliferation assays were performed similarly to the HFF proliferation assays with modifications. Confluent HepG2 cells were harvested by treatment with 1 × trypsin–EDTA and diluted in RPMI-1640 supplemented with 10% (vol/vol) fetal bovine serum. Assays were set up in 96-well microtiter plates with 20,000 cells per well. Puromycin (10 µM) was used as a non-proliferation control, and 0.1% (vol/vol) DMSO served as a 100% proliferation control. The plates were incubated at 37°C in a CO_2_ incubator for 72 h, and the Incucyte Live-Cell Analysis System (Essen BioScience) was used to capture cellular changes and assess cell growth in each well over time as described above for the HFF proliferation assays. The bottom of the curve was constrained to 0 when fitting for HepG2 proliferation assays using GraphPad Prism.

### Preparation of *P. falciparum* lysates

Parasite lysates were prepared from saponin-isolated mature trophozoite-stage parasites as described by Saliba et al*.* (14), with modifications. Briefly, a culture with predominantly trophozoite stage parasites was thoroughly mixed with saponin (0.05% [wt/vol] final concentration) and centrifuged at 2,000 × *g* for 8 min at 4°C. The pelleted parasites were then transferred to a 1.5 mL tube and washed three times (16,000 × *g*, 1 min each time) with cold malaria saline (125 mM NaCl, 5 mM KCl, 25 mM HEPES, 20 mM glucose, 1 mM MgCl_2_, pH 7.1). Subsequently, washed saponin-isolated parasites were suspended in 1 mL of 10 mM Tris/Cl, pH 7.4, followed by trituration (10 times) through a SafetyGlide injection needle (25-gauge). The lysates were then centrifuged at 16,000 × *g* for 30 min at 4°C, and the supernatant was transferred to a new tube. The concentration of the lysates was then determined using the Bradford reagent.

### [^14^C]pantothenate phosphorylation assay

Phosphorylation of [^14^C]pantothenate was determined using a combination of zinc sulphate (ZnSO_4_) and barium hydroxide (Ba(OH)_2_) (Somogyi reagent) as previously described (78). In the 125 min time course, each reaction (650 µL) contained a final concentration of 50 mM Tris-Cl (pH 7.4), 5 mM ATP, 5 mM MgCl_2_, 2 µM (0.1 µCi/mL) [^14^C]pantothenate, and 60 µg of total parasite lysate. The compounds were added at their 10× antiplasmodial IC_50_ values or at various concentrations to generate dose–response curves. Mixtures were incubated at 37°C throughout the experiment, and reactions were initiated by the addition of the lysates. A reaction with identical components to the test reactions but lacking the parasite lysate was prepared to serve as a no-phosphorylation negative control, and a reaction with DMSO vehicle control (0.2% vol/vol) served as a positive control. At pre-determined time points, 50 µL of the reaction mixture was transferred in triplicate to wells of a 96-well filter plate with 0.2 µm hydrophilic polyvinylidene fluoride (PVDF) membrane filter (Corning), which had been pre-loaded with 50 µL of 150 mM Ba(OH)_2_. The reaction mixtures were thoroughly mixed with the Ba(OH)_2_ in the wells to precipitate the enzyme and terminate the phosphorylation reaction. After all reactions had been terminated, phosphorylated compounds in each well were precipitated by the addition of 50 µL of 150 mM ZnSO_4_ to generate the Somogyi reagent. The plate was then washed twice with deionized H_2_O and once with 95% (vol/vol) ethanol using a vacuum manifold to remove the non-phosphorylated [^14^C]pantothenate and was subsequently dried overnight at 37°C in a non-humidified incubator. The following day, 30 µL of Microscint-O (PerkinElmer) was added to each well, and the plate was sealed on the top and bottom with TopSeal-A Plus sealing film (PerkinElmer). A Packard Microplate Scintillation and Luminescence Counter was used to measure the radioactivity in each well immediately after the plate had been sealed. To determine the total radioactivity in each phosphorylation assay, 50 µL of each reaction mixture was added to the wells of an OptiPlate-96 microplate (PerkinElmer) in triplicate and was then thoroughly mixed with 150 µL of Microscint-40 (PerkinElmer), and the radioactivity was measured in the same manner. Phosphorylation of [^14^C]pantothenate, calculated as a percentage of the radioactivity measured in the test wells relative to the total radioactivity in the phosphorylation assay, was then plotted against the reaction time. Each data set was analyzed by linear regression, from which the phosphorylation rate of each reaction was then determined and expressed as a percentage of the phosphorylation rate of the DMSO-positive control reaction.

### *Hs*PanK protein cloning, expression, and purification

The gene encoding the catalytic core domain of human PanK3 (amino acid residues 12–368) was synthesized and cloned into the pET28a(+) expression vector using the NdeI and BamHI restriction sites to produce an N-terminal His6-tagged protein (Bio Basic Inc.). The plasmid was transformed into *E. coli* BL21(DE3) cells, which were cultured in Terrific Broth (TB) medium containing 50 µg/mL kanamycin at 37°C. When the culture reached an optical density at 600 nm (OD_600_) of 0.6, protein expression was induced by adding 1.0 mM isopropyl β-D-thiogalactoside (IPTG), followed by incubation at 18°C for 17–18 h. The cells were then harvested and resuspended in lysis buffer (20 mM Tris-HCl, pH 7.5, 500 mM NaCl, 5 mM imidazole, 5% [vol/vol] glycerol) supplemented with 1 mM PMSF (phenylmethylsulfonyl fluoride) and lysozyme (5 mg/g of the cell pellet). The suspension was then sonicated on ice, and the resulting lysate was centrifuged twice at 54,000 × *g* for 30 min at 4°C.

The clarified supernatant was incubated with Ni-NTA resin (Cytiva) pre-equilibrated with lysis buffer containing 5 mM imidazole at 4°C. After binding, the resin was washed with lysis buffer containing 30 mM imidazole, then eluted with lysis buffer containing 250 mM imidazole. The eluted *Hs*PanK3 protein was dialyzed against storage buffer (20 mM Tris-HCl, pH 7.5, 200 mM NaCl, 5% [vol/vol] glycerol) and concentrated. The purified protein was stored at −80°C. Protein purity was assessed using SDS-PAGE and deemed >50% (Fig. S4).

### *Hs*PanK kinetic assays

The enzyme assay was adapted from a previously described method (103), which links the production of ADP to the consumption of NADH through the enzymatic activities of pyruvate kinase and lactic dehydrogenase. Reactions were conducted in 96-well microtiter plates, and the decrease in NADH concentration was measured at 340 nm at 30 s intervals after incubation at 30°C. Each reaction mixture (200 µL) contained the desired compound (substrate) at variable concentrations (0–2,000 µM), ATP (1.5 mM), NADH (0.3 mM), phospho(enol)pyruvate (2.0 mM), MgCl_2_ (10 mM), KCl (20 mM), a commercial pyruvate kinase/lactic dehydrogenase mixture (12 units, Sigma-Aldrich), and pantothenate kinase (0.48 µM) in 50 mM Tris–HCl buffer (pH 7.6). The reaction was initiated with the addition of ATP. The kinetic parameters were determined by fitting the rate data into the Michaelis–Menten equation using GraphPad Prism 8.0 with nonlinear regressions. Data were collected from three independent experiments, each performed in triplicate to verify the trends and optimize the selected concentrations. The kinetic parameters were calculated from the most complete triplicate set of data, and errors represent SEM. For cases where substrate inhibition was suspected, the same conditions were used, but the data were fitted using the built-in Equation 1 (Y = V_max_*X/(*K*_m_ +X*(1 + X/*K*_i_)) from GraphPad Prism, where X and Y represent substrate concentration and enzyme activity, respectively, V_max_ is the maximum enzyme activity in the same units as Y, and the *K*_m_ and *K*_i_ are the Michaelis–Menten and the inhibitor constants, respectively, both in the same units as X. Equation 1 is based on Equation 5.44, in RA Copeland, Enzymes, 2nd edition, Wiley, 2000.

### Compound synthesis and characterization

Details of chemical synthesis as well as compound characterization are shown in Supporting Information 2. NMR spectra are also included starting on page S40.

### Statistics

Statistical analysis was carried out using unpaired, two-tailed Student’s t-test using GraphPad Prism 10.0.

## SUPPLEMENTAL MATERIAL

Supplemental figures aac.00331-25-s0001.pdf Fig. S1 to S5.

Supplemental material aac.00331-25-s0002.pdf Additional chemistry methodology and data.

ASM does not own the copyrights to Supplemental Material that may be linked to, or accessed through, an article. The authors have granted ASM a non-exclusive, world-wide license to publish the Supplemental Material files. Please contact the corresponding author directly for reuse.
