# Supplementary material for: Thiazole substitution of a labile amide bond—a new option toward antiplasmodial pantothenamide-mimics
Source: Antimicrob Agents Chemother. 2025 Aug 13;69(9):e00331-25. doi: 10.1128/aac.00331-25 (PMC12406685; doi:10.1128/aac.00331-25)
Supplement: Supplemental figures — Fig. S1 to S5. [file aac.00331-25-s0001.pdf]

SUPPORTING INFORMATION 1

Supplementary biological results

Thiazole substitution of a labile amide bond - a new option towards antiplasmodial pantothenamide-mimics

Xiangning Liu[1], Annica Chu[2], Mina Nekouei[2], Chunling Blue Lan[2], Alexandre Pierret[2], Karine Auclair[2*], Kevin J. Saliba[1*]

[1]Research School of Biology, Australian National University, Canberra, ACT, AUSTRALIA

[2]Department of Chemistry, McGill University, Montreal, Quebec, CANADA H3A 0B8

*To whom correspondence should be addressed: k.auclair@mcgill.ca or kevin.saliba@anu.edu.au

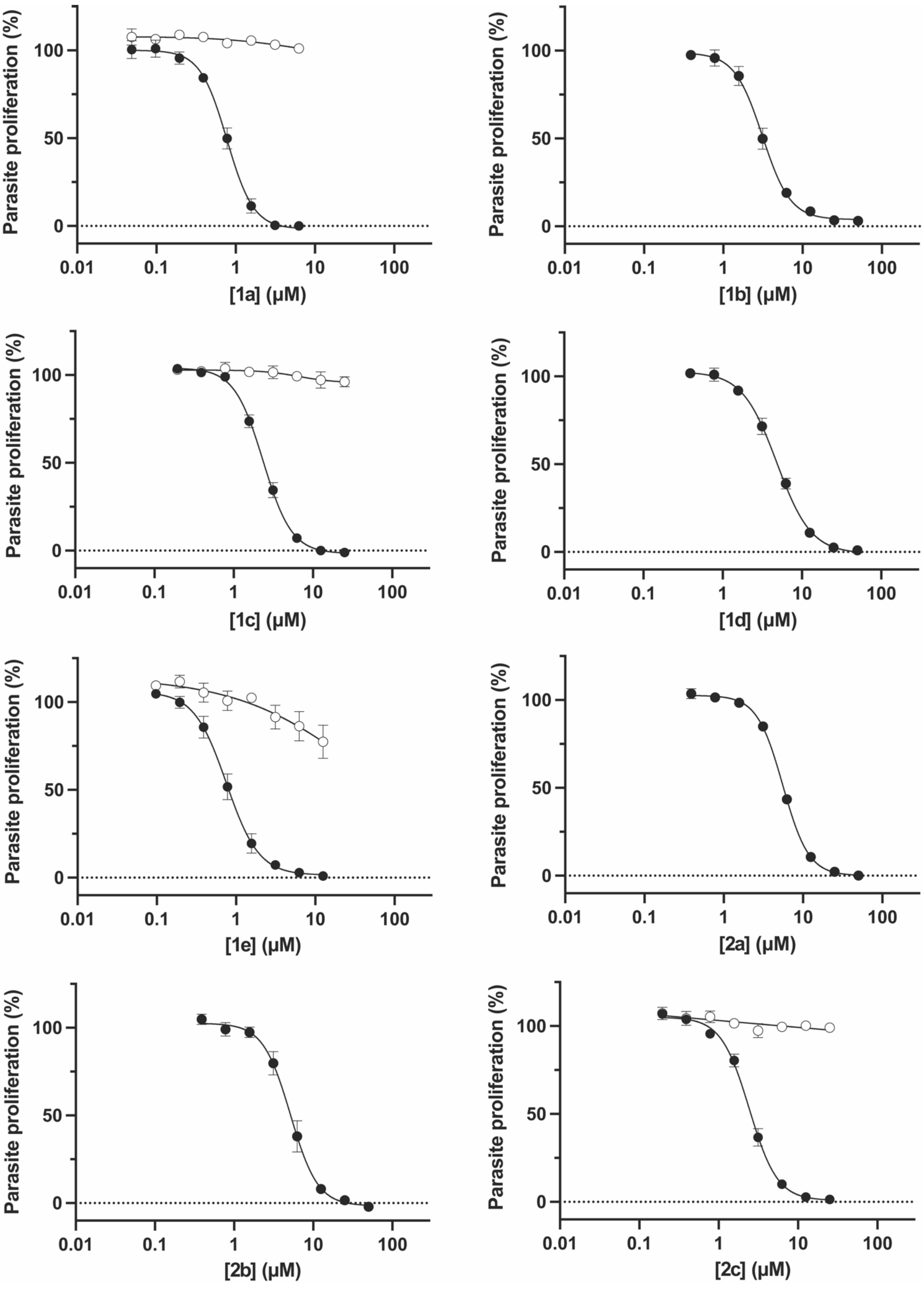
Parasite proliferation (%)
100
50
0
0.01
0.1
1
10
100
[1a] (μM)
[1b] (μM)
[1c] (μM)
[1d] (μM)
[1e] (μM)
[2a] (μM)
[2b] (μM)
[2c] (μM)

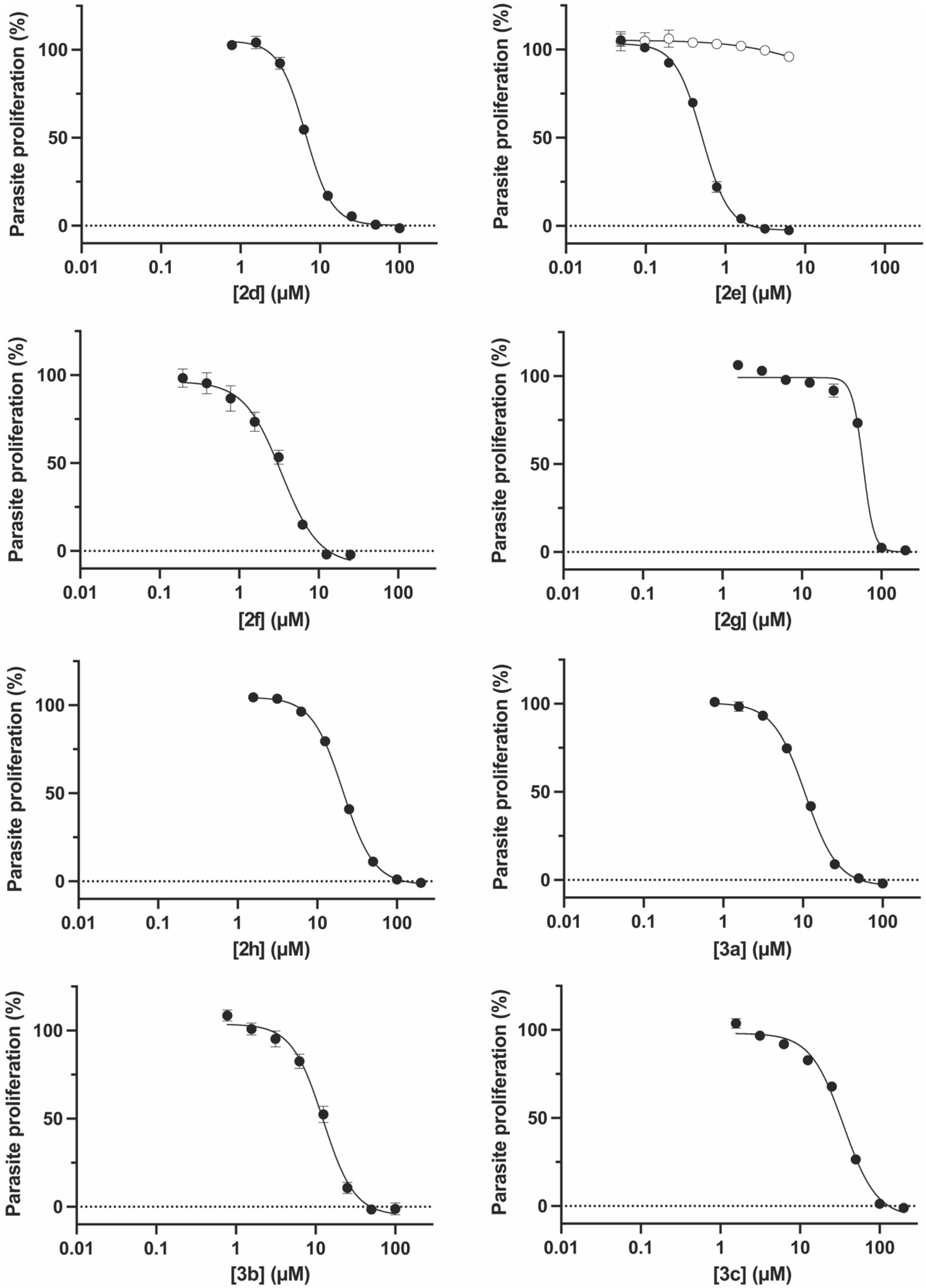
Parasite proliferation (%)
100
50
0
0.01
0.1
1
10
100
[2d] (μM)
[2e] (μM)
[2f] (μM)
[2g] (μM)
[2h] (μM)
[3a] (μM)
[3b] (μM)
[3c] (μM)

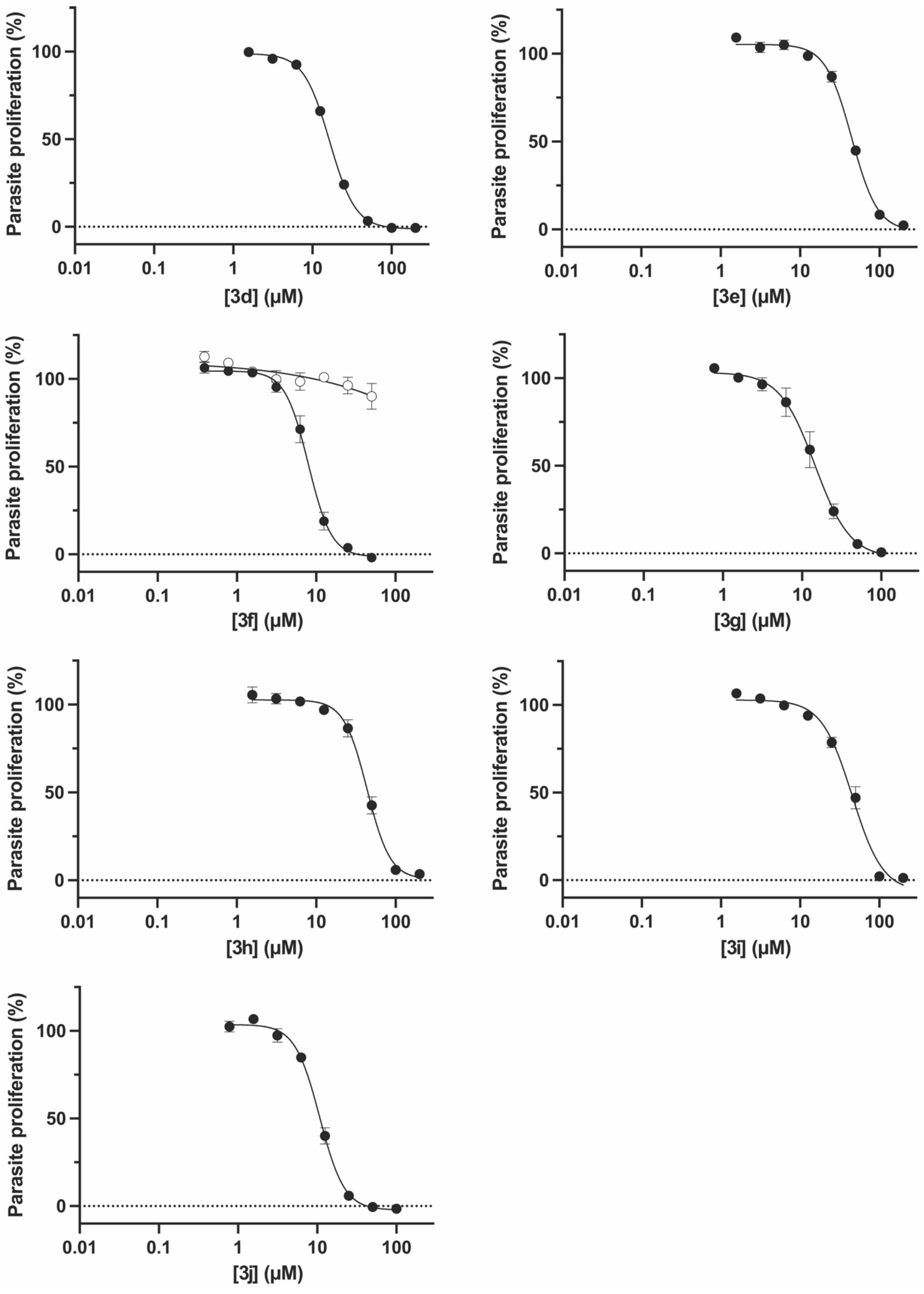
Parasite proliferation (%)
100
50
0
0.01
0.1
1
10
100
[3d] (µM)
[3e] (µM)
[3f] (µM)
[3g] (µM)
[3h] (µM)
[3i] (µM)
[3j] (µM)

Figure S1. The effect of thiazole-bearing pantothenamide mimics on the proliferation of *P. falciparum*. The effect of the compounds on the proliferation of *P. falciparum* 3D7 parasites in the presence of 1 μM (black circles) or, if carried out, 100 μM pantothenate (white circles). Values are averaged from three independent experiments, each carried out in triplicate. Error bars represent SEM and where not visible, are smaller than the symbols.

A

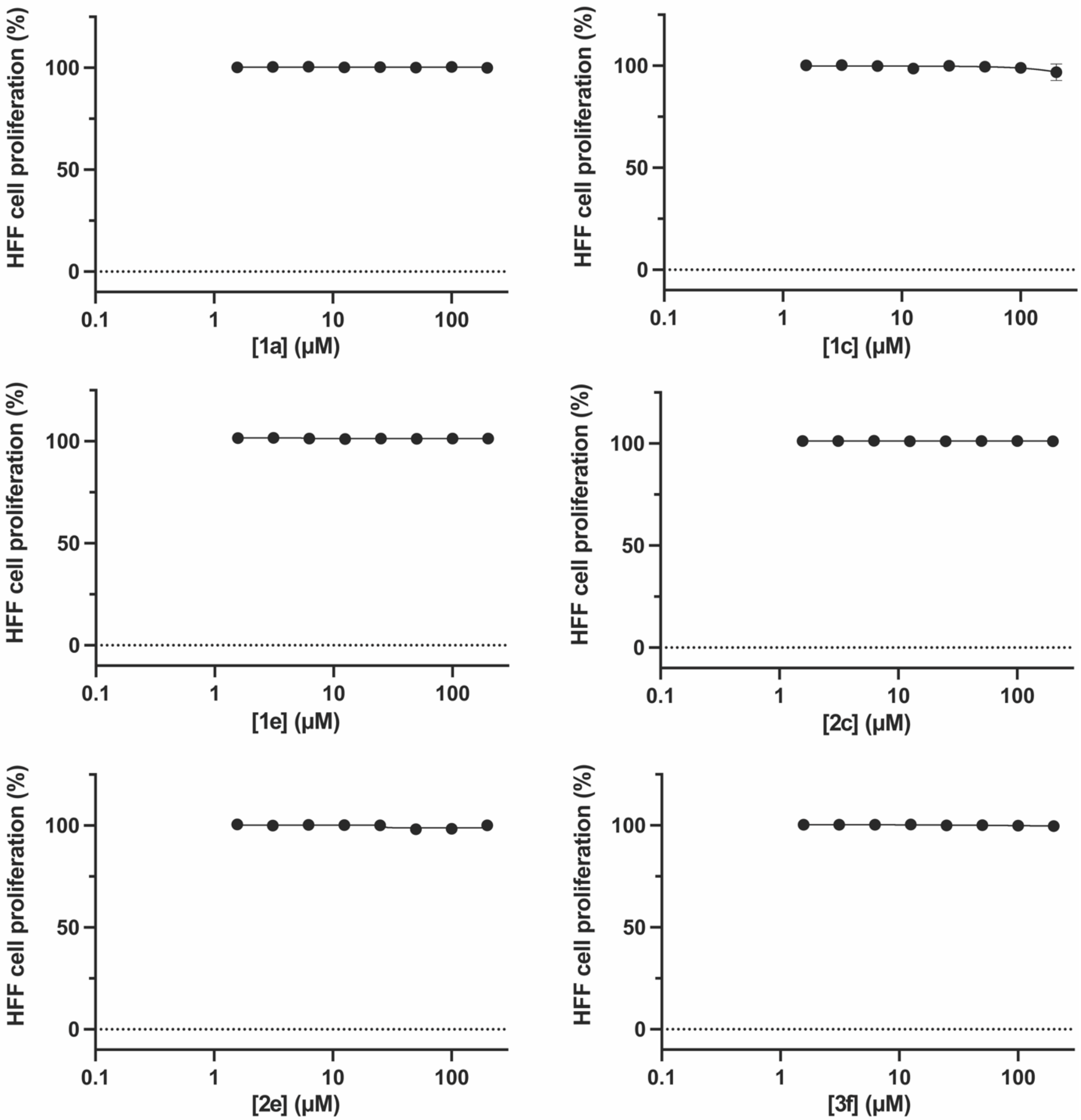
HFF cell proliferation (%)
100
50
0
0.1
1
10
100
[1a] (µM)
[1c] (µM)
[1e] (µM)
[2c] (µM)
[2e] (µM)
[3f] (µM)

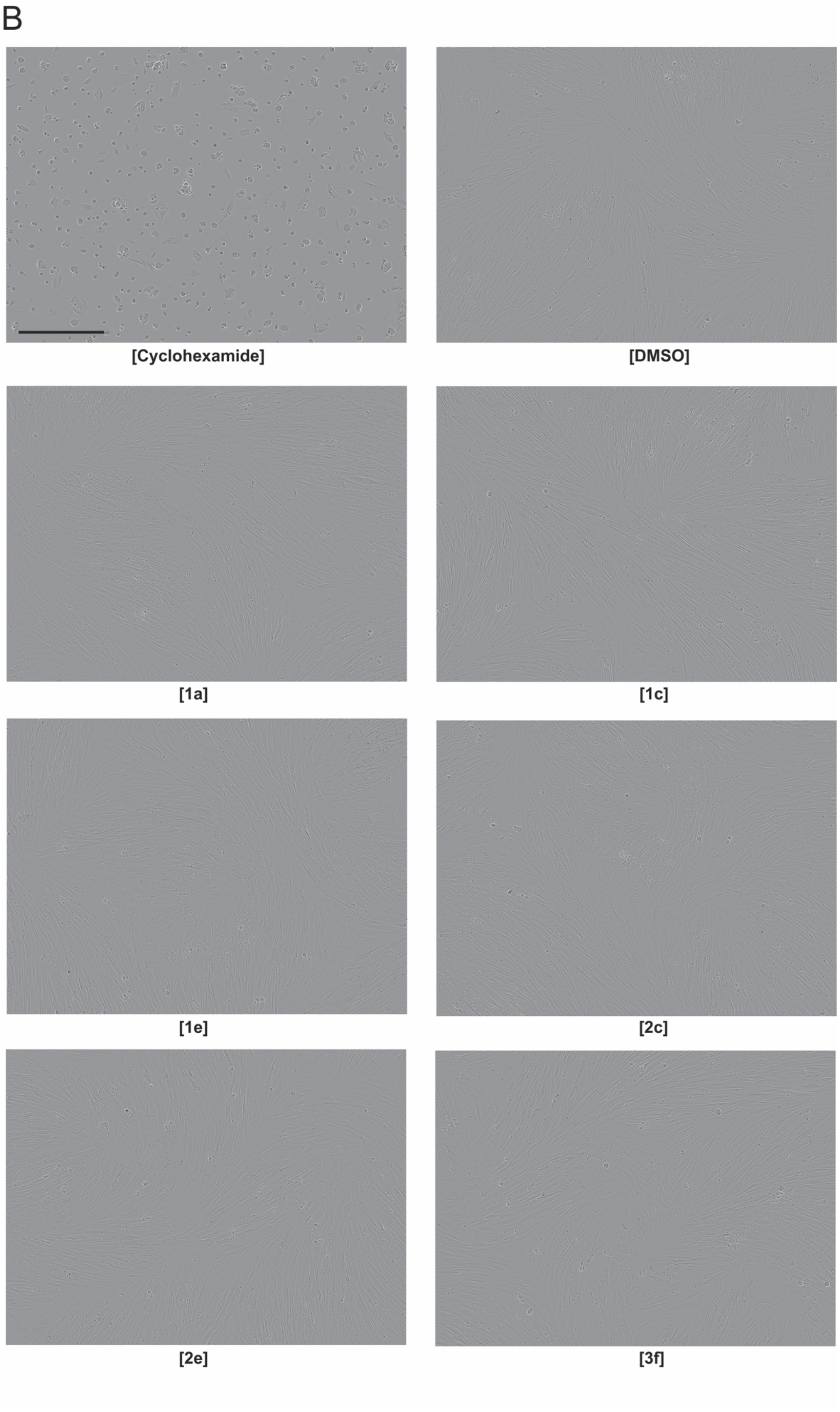
B
[Cyclohexamide]
[DMSO]
[1a]
[1c]
[1e]
[2c]
[2e]
[3f]

Figure S2. The effect of thiazole-bearing pantothenamide mimics on the proliferation of HFF cells. The effect of the compounds on the proliferation of HFF cells are shown in A. Values are averaged from 2 independent experiments, each carried out in triplicate. Error bars represent range/2 and where not visible, are smaller than the symbols. Microscopy images of HFF cells incubated with a concentration of 200 µM compounds are shown in B. The effect of 10 µM cyclohexamide and DMSO (vehicle control) on the proliferation of HFF cells is also shown (top two images). Images are captured by Incucyte® Live-Cell Analysis System and are representative of 2 independent experiments. Scale bar represents 400 µm.

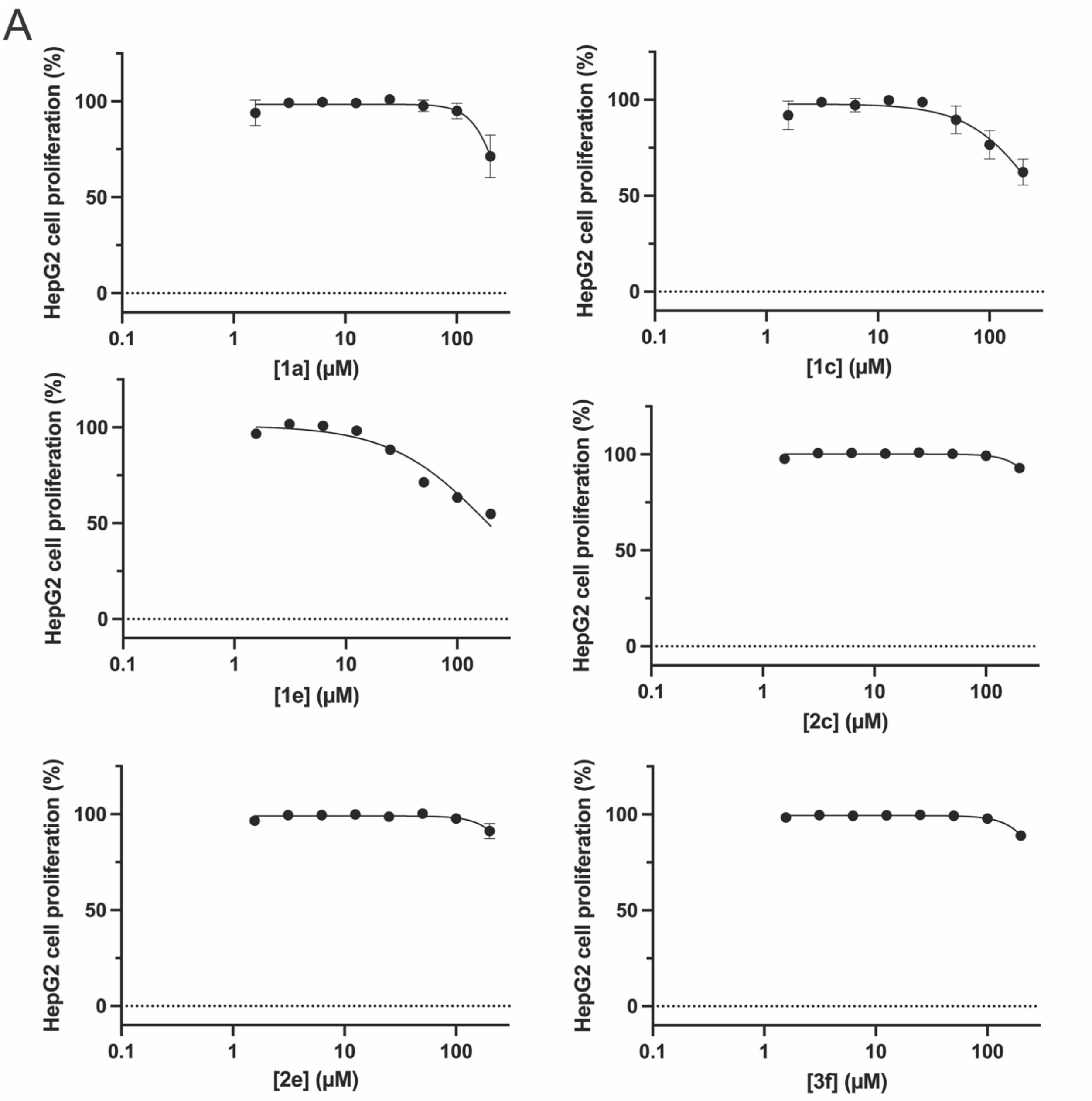
A
HepG2 cell proliferation (%)
100
50
0
0.1
1
10
100
[1a] (μM)
[1c] (μM)
[1e] (μM)
[2c] (μM)
[2e] (μM)
[3f] (μM)

B

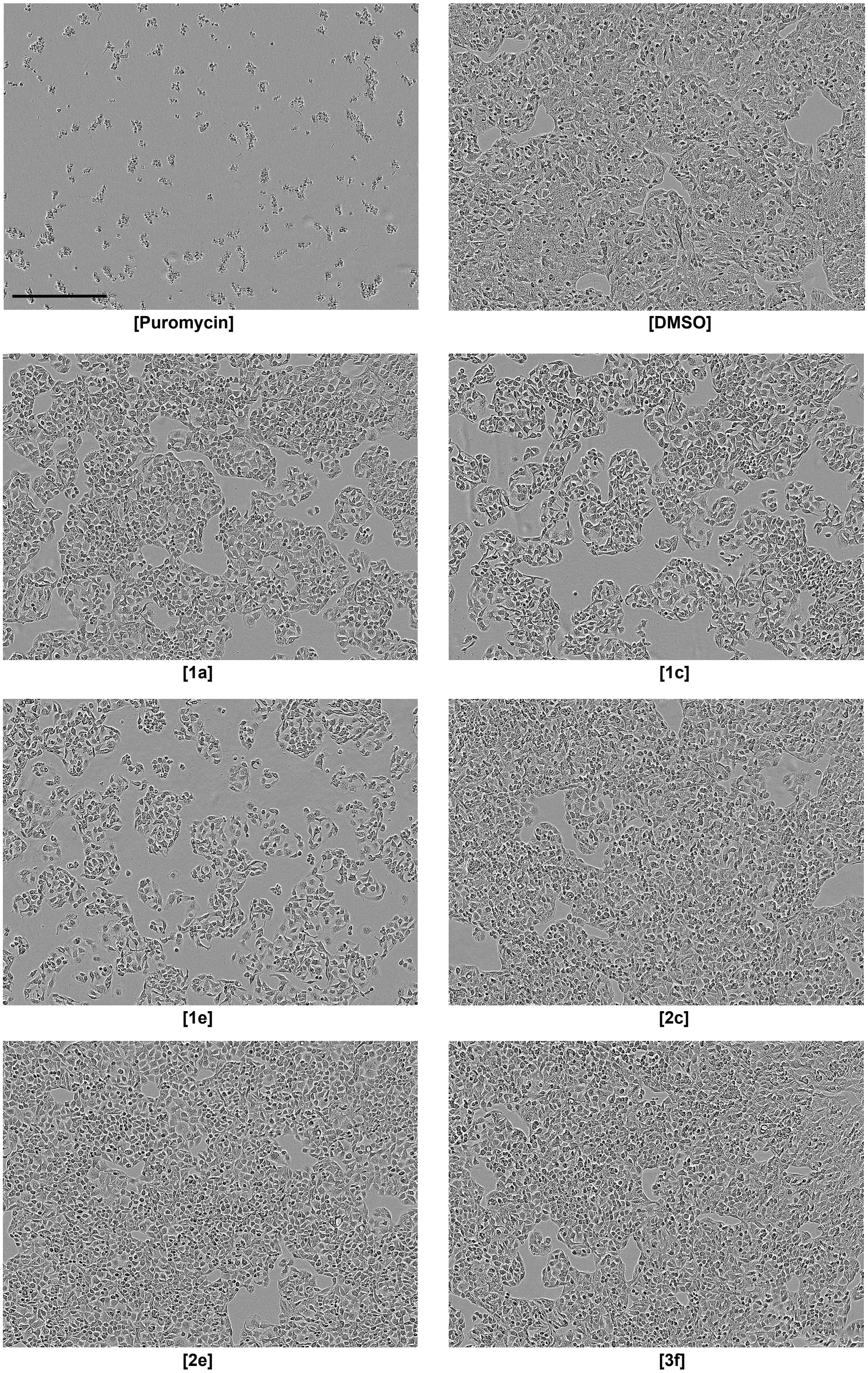

Figure S3. The effect of thiazole-bearing pantothenamide mimics on the proliferation of HepG2 cells. The effect of the compounds on the proliferation of HepG2 cells are shown in A. Values are averaged from 2 independent experiments, each carried out in triplicate. Error bars represent range/2 and where not visible, are smaller than the symbols. Microscopy images of HepG2 cells incubated with a concentration of 200 μM compounds are shown in B. The effect of 10 μM puromycin and DMSO (vehicle control) on the proliferation of HepG2 cells is also shown (top two images). Images are captured by Incucyte® Live-Cell Analysis System and are representative of 2 independent experiments. Scale bar represents 400 μm.

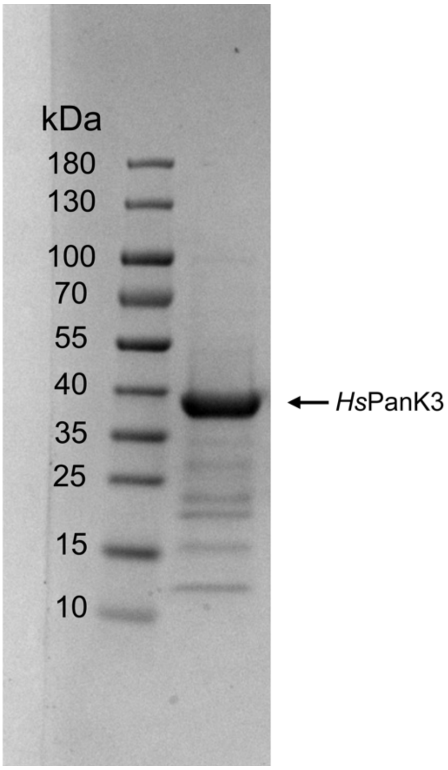

Figure S4. SDS-PAGE gel analysis of eluted *Hs*PanK protein following Ni-NTA purification. The right lane contains an aliquot of the purified protein (predicted weight of 41.6 kDa including the N-terminal His_6-tag and a thrombin cleavage site that have not been removed). The protein ladder is shown on the left, with molecular weights indicated.

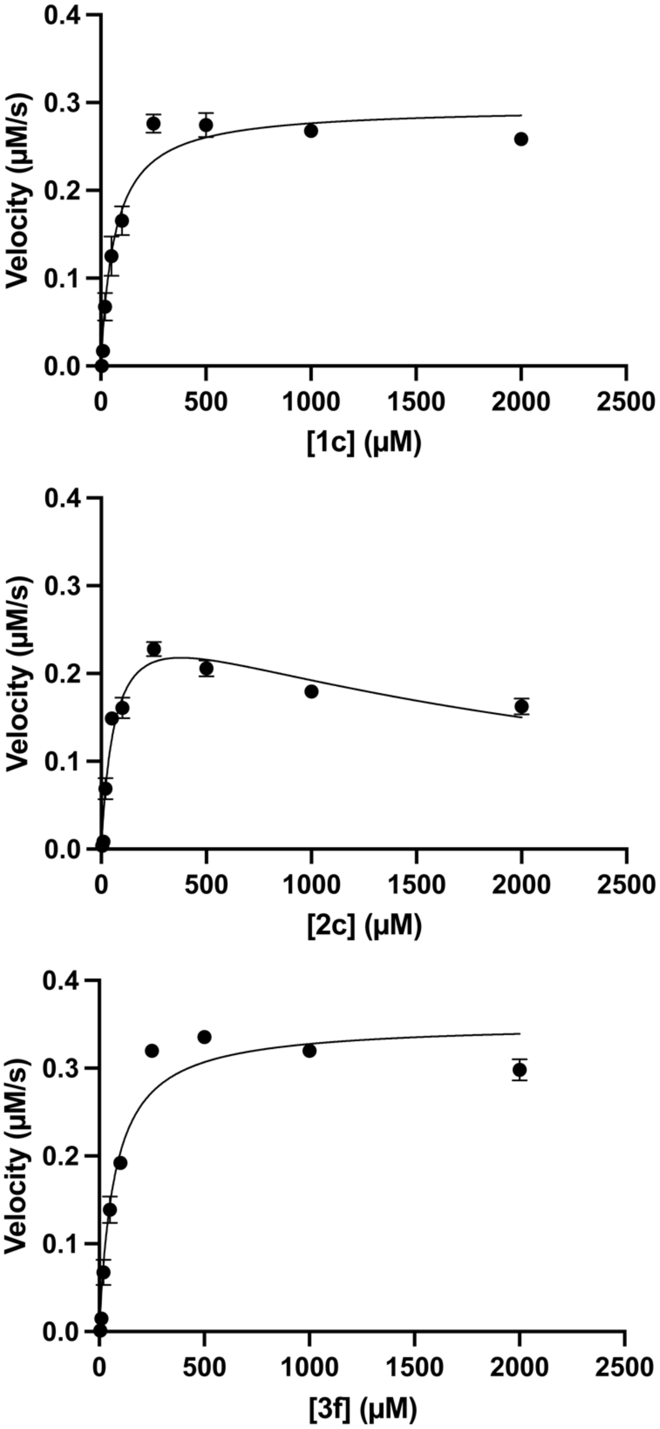

Figure S5. Kinetics plots for *Hs*PanK3 in the presence of 1c, 2c or 3f. Velocities were determined as a function of compound concentration. For **1c** and **3f**, the data points were fit to the Michaelis–Menten nonlinear regression equation to determine kinetic parameters. For **2c**, Equation 1, which accounts for substrate inhibition, was used to fit the data. Data are averaged from 3 independent experiments, each performed in triplicate. Error bars represent SEM and are not visible if smaller than the symbols.
